# New Benzamides as Multi-Targeted Compounds: A Study on Synthesis, AChE and BACE1 Inhibitory Activity and Molecular Docking

**DOI:** 10.3390/ijms241914901

**Published:** 2023-10-04

**Authors:** Danuta Drozdowska, Dawid Maliszewski, Agnieszka Wróbel, Artur Ratkiewicz, Michał Sienkiewicz

**Affiliations:** 1 Department of Organic Chemistry, Medical University of Białystok, Mickiewicza Street 2A, 15-222 Białystok, Poland; dawidmaliszewski.dm@gmail.com (D.M.); agnieszkawrobel9@gmail.com (A.W.); 2 Department of Physical Chemistry, Faculty of Chemistry, University of Białystok, Ciołkowskiego 1K Street, 15-245 Białystok, Poland; artrat@uwb.edu.pl (A.R.); mikes@uwb.edu.pl (M.S.)

**Keywords:** Alzheimer’s disease, AChE inhibitors, BACE1 inhibitors, benzamides, dual inhibitors

## Abstract

The synthesis of eleven new and previously undescribed benzamides was designed. These compounds were specifically projected as potential inhibitors of the enzymes acetylcholinesterase (AChE) and β-secretase (BACE1). N,N′-(1,4-phenylene)bis(3-methoxybenzamide) was most active against AChE, with an inhibitory concentration of AChE IC_50_ = 0.056 µM, while the IC_50_ for donepezil was 0.046 µM. This compound was also the most active against the BACE1 enzyme. The IC_50_ value was 9.01 µM compared to that for quercetin, with IC_50_ = 4.89 µM. Quantitative results identified this derivative to be the most promising. Molecular modeling was performed to elucidate the potential mechanism of action of this compound. Dynamic simulations showed that new ligands only had a limited stabilizing effect on AChE, but all clearly reduced the flexibility of the enzyme. It can, therefore, be concluded that a possible mechanism of inhibition increases the stiffness and decreases the flexibility of the enzyme, which obviously impedes its proper function. An analysis of the H-bonding patterns suggests a different mechanism (from other ligands) when interacting the most active derivative with the enzyme.

## 1. Introduction

Alzheimer’s disease (AD) is a neurodegenerative disease characterized by the progressive loss of memory, which is associated with other cognitive deficits. The complex pathophysiology of this disease includes impaired neurotransmission, the aggregation of pathological proteins, and increased oxidative stress, among others. Thus far, drugs that can act even on one of these abnormalities associated with Alzheimer’s disease have not been successfully found in the search for an effective treatment. Therefore, multi-targeted drugs (MTDs) that act simultaneously on several molecular targets seem to be a better option, although this approach also has some limitations [1]. There are various hypotheses regarding the causes of AD, among which cholinesterase activity still remains a key biological target in the search for AD therapy [2]. A decrease in levels of the neurotransmitter acetylcholine (ACh) in the brain is one of the leading causes of AD. This is partly due to the increased activity of acetylcholinesterase (AChE), which is the enzyme responsible for its breakdown [3,4]. It was also confirmed that the inhibition of β-amyloid aggregation could modify the course of AD. Insoluble Aβ aggregates lead to plaque deposition and neurodegeneration. The ẞ-site APP cleaving enzyme-1 (BACE1) is involved in the rate-limiting step of the cleavage process of the amyloid precursor protein (APP), leading to the generation of the neurotoxic amyloid β (Aβ) protein, which is the next attractive target for the treatment of AD [5].

Investigations to obtain new multi-target potential drugs for Alzheimer’s disease have been carried out very intensively [6,7,8].

Among the many investigated structures, a lot of derivatives are benzamide compounds. Such compounds are useful building blocks in organic synthesis and enable the introduction of different substituents, allowing for a detailed structure–activity analysis. Amide bond formation is an important transformation in organic synthesis. These bonds are often present in many active derivatives and are characterized by a variety of pharmacological activities, e.g., anti-inflammatory, analgesic, antimicrobial, or anticancer activities. The amide group is also a useful intermediate for the synthesis of various biologically active molecules [9]. Aromatic amide compounds are stable and relatively easy to obtain from commercially available substrates. Our team has many years of experience in synthesizing and studying the activity of various substituted benzamides [10].

**Figure 1:**
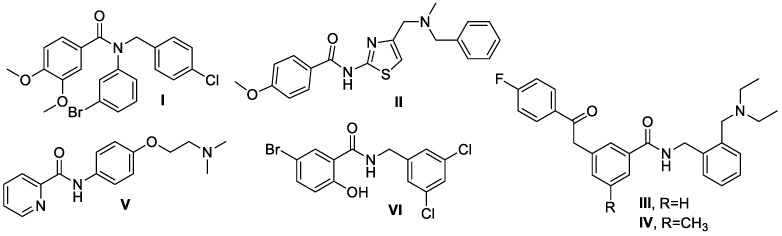
Structures of dual AChE and BuChE inhibitors.

Figure 1 presents benzamide structures published in recent years showing activity against different molecular targets of Alzheimer’s disease. Koca et al. synthesized novel benzamide derivatives and examined them as inhibitors against AChE and butyrylocholinesterase (BuChE). The most effective inhibitor was compound **I** for both AChE and BuChE, with IC_50_ values of 1.57 and 2.85 μM, respectively [11]. Also, substance **II** synthesized by Kilic’s team was found to be a dual cholinesterase inhibitor (AChE IC_50_ = 1.47 and BuChE IC_50_ = 11.40 μM) [12]. *N*-benzyl benzamide inhibitors investigated by Du’s research group seemed promising compounds with drug-like properties—compounds **III** and **IV** exhibited very strong inhibitory effects on BChE, with IC_50_ values of 0.08 and 0.039 nM, respectively [13]. Compound **V,** studied by the Gao scientific team, revealed the most potent AChE inhibitory activity (IC_50_: 2.49  ±  0.19 μM) and the highest selectivity against AChE over BuChE [14]. Among the 36 derivatives of 5-bromosalicylic acid presented by Kratky and others, compound **VI** showed the highest inhibitory potency of AChE activity (33.13 ± 0.47 μM) [15].

Cholinesterase inhibitors are the main class of drugs that are currently used to treat Alzheimer’s disease, but they have limited activity, and it is necessary to supplement therapy by combining them with other classes of drugs. For this reason, it seems relevant to investigate the activity of new compounds against an enzyme that has a completely different action and a different molecular target, i.e., BACE1. It has been observed that when amyloidogenic β-secretase is inhibited, the production of Aβ peptide and brain amyloid-β (Aβ) accumulation is reduced. Therefore, the discovery of new small bioactive molecules that potentially reach the brain and inhibit BACE1 is a new and potentially important research direction for AD therapy.

**Figure 2:**
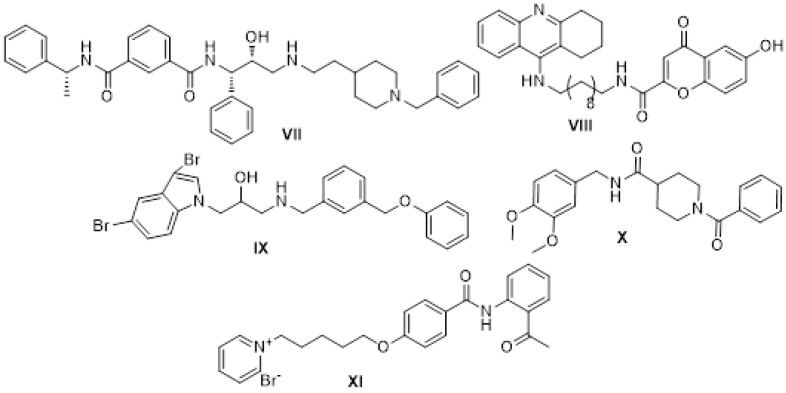
Structures of dual AChE and BACE1 inhibitors.

In recent years, a number of compounds have been investigated that can inhibit both AChE and ẞ-secretase (BACE1): an enzyme involved in cutting the amyloid beta precursor protein (APP). The studied structures have included aromatic amide compounds. The structures of selected benzamides, active against both enzymes, are shown in Figure 2.

Derivative **VII**, designed as a structure combining the phthalamide and donepezil fragment by Zhu and al., showed good activity against both enzymes (AChE IC_50_ = 1.83 and BACE1 IC_50_ = 0.57 μM) [16]. Molecule **VIII**, containing a tacrine fragment in the structure obtained by the team of Fernandez-Bachiller, also showed potent dual inhibition against human AChE and BACE-1 (IC_50_ = 8.0 nM for AChE and IC_50_ = 2.8 mM for BACE-1) [17]. Dominguez and co-authors obtained compound **IX** through a computer-aided design, which showed a marked dual inhibitory activity against AChE (IC_50_ = 9.1 mM) and BACE-1 (IC_50_ = 2.5 mM) [18]. Notably, compound **X**, containing a benzophenone core presented by Gabr’s group, has shown a strong inhibition of AChE (IC_50_ = 4.11 nM) and BACE-1 (IC_50_ = 18.30 nM) in humans [19]. A detailed review of natural and synthetic compounds with dual inhibitory properties against both AChE and BACE-1, as well as a comprehensive structure–activity relationship (SAR) analysis of synthetic compounds, was presented by Ferreira and co-authors [20]. Among the many examples, Peng’s team designed the highly interesting compound **XI**, with a positively charged pyridine ring, which was found to be a potent inhibitor of BACE1 (IC_50_ = 0.31 μM) with an activity against both cholinesterases in nanomolar ranges (hAChE Ki = 81 nM, hBuChE Ki = 93 nM) [21].

We present here only some examples of benzamide derivatives with interesting activity and potential utility in the treatment of Alzheimer’s disease. Many series of new multi-target potential AD drugs have been developed and described [22,23,24].

**Figure 3:**
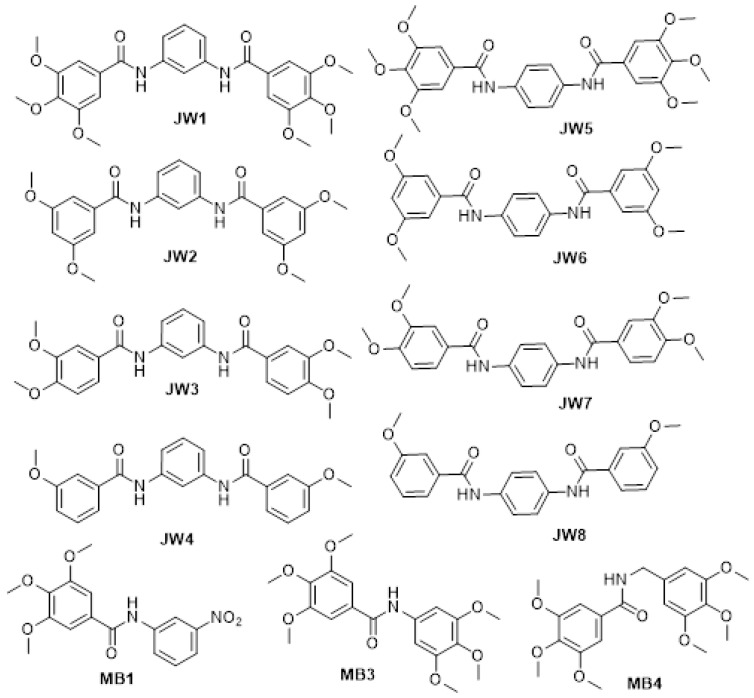
Structures of new benzamides.

Based on our extensive experience in synthesizing and studying the anticancer activity of various benzamide compounds [9] and reports summarizing the diversity of activities that such derivatives show [25], we designed the synthesis of eleven new compounds to investigate their potential utility in AD therapy. The advantages of this type of derivative include easy synthesis, the availability and low cost of substrates, and the stability of products. Figure 3 presents a series of newly synthesized benzamide compounds. All of them have been shown to have an inhibitory effect on acetylcholinesterase as well as ẞ-secretase, confirming their potential to treat Alzheimer’s disease. 

## 2. Results and Discussion

### 2.1. Chemistry

Amide bond formation is an important transformation in organic synthesis. These bonds are often present in many active derivatives and are characterized by a variety of pharmacological activities, e.g., anti-inflammatory, analgesic, antimicrobial, or anticancer activities. For the preparation of new benzamides, we used a conventional solution-based synthesis method. 

The compounds **JW1**–**JW8** were obtained using round bottom flasks in which phenylenediamine was solved in dichloromethane (DCM) with N,N-diisopropylethylamine (DIPEA). The mixture was cooled in an ice bath. Then, solutions of acyl chlorides in dry DCM were added dropwise under an atmosphere of inert gas formed. White precipitations were formed during reactions. The amount of o-phenylenediamine was monitored using TLC (DCM/methanol 9:1) stained with DMAB. The reactions were stopped after the observed substrates were consumed. The white precipitations were filtered under a vacuum and washed three times with 10% HCl and three times with 10% NaHCO_3_. The obtained products were dried under a vacuum. 

The same procedure was used to obtain the compounds **MB1**, **MB3**, and **MB4**. The solid states were crystalized from the boiling mixture of ethyl acetate and hexane. The products were filtrated and dried under a vacuum. 

All structures and identities of the obtained compounds were confirmed using ^1^H and ^13^C NMR spectroscopy and mass spectrometry (see Supplementary Materials).

### 2.2. In Vitro AChE and BACE1 Inhibitory Activity

The ability of the resulting benzamides to inhibit AChE and BACE1 activity was assessed in vitro via methods described previously [26] using standards (donepezil, tacrine, and quercetin). Experiments were repeated three times; the calculated inhibitory concentration of half the enzymatic activity, i.e., IC_50_ (µM), is included in Table 1. All compounds were characterized by their activity against the enzymes tested with calculated IC_50_ values, ranging from 0.056 to 2.57 μM for AChE and from 9.01 to 87.31 μM for BACE1. However, neither compound showed higher activity against AChE than the reference compound donepezil (IC_50_ = 0.046 μM). To block BACE1 activity, compound **JW8** had the best effect, with IC_50_ = 9.01 μM, but it showed half the inhibitory activity of comparative quercetin (IC_50_ = 4.89 μM).

### 2.3. Molecular Docking

In order to obtain a better insight into the mechanism of ligand interaction with AChE/BACE1 receptors, affinities were tested in silico using a molecular docking simulation. As detailed in Section 3.3, the docking protocol was verified by removing the inhibitor from both structures (i.e., PDB:73EH and PDB:5HU1), redocking, and computing the root mean square deviations (RMSDs). The resulting affinities are posted in Table 1 above. It is known that the docking score can be related to the size of the molecule. Due to the larger number of interactions, larger ligands are likely to show a higher docking affinity. Accordingly, a value normalized by the size of the compound, called ligand efficiency (LE) [27], can also be considered:

(1) $$LE=-\frac{Docking\;affinity}{Number\;of\;non-hydrogen\;atoms\;in\;the\;molecule}$$

The best affinities were exhibited by the reference ligands, namely donepezil (−11.6 kcal/mol) for AChE and verubecestat (−8.9 kcal/mol) for BACE1. However, for AChE, three of the new derivatives (**JW4**, **JW7**, and **JW8**) showed affinities only slightly higher (−11.2, −10.8, and −11.2 kcal/mol, respectively), while ligand efficiency was almost the same as donepezil. It is worth noting that tacrine, with a poor docking result of −8.9 kcal/mol, shows the best LE, which is much higher than that of the other compounds of concern. On the other hand, quercetin showed a good affinity for BACE1 (−8.4 kcal/mol), second only to verubecestat (−8.9 kcal/mol), while LE was definitely the best here. Of the new derivatives, the best affinities were shown by **JW4** (−8.2 kcal/mol), **JW7** (−8.1 kcal/mol), and **JW8** (−8.1 kcal/mol). However, LE values were noticeably poorer than reference substances. In general, it can be concluded that the proposed compounds show inhibitory activity that is comparable to drugs already in use, and the docking results show similar trends to experimental IC_50_ measurements. The most promising ligand is **JW8**, but **JW4** and **JW7** are also of interest. For this reason, these compounds continue to be employed in further discussion.

**Figure 4:**
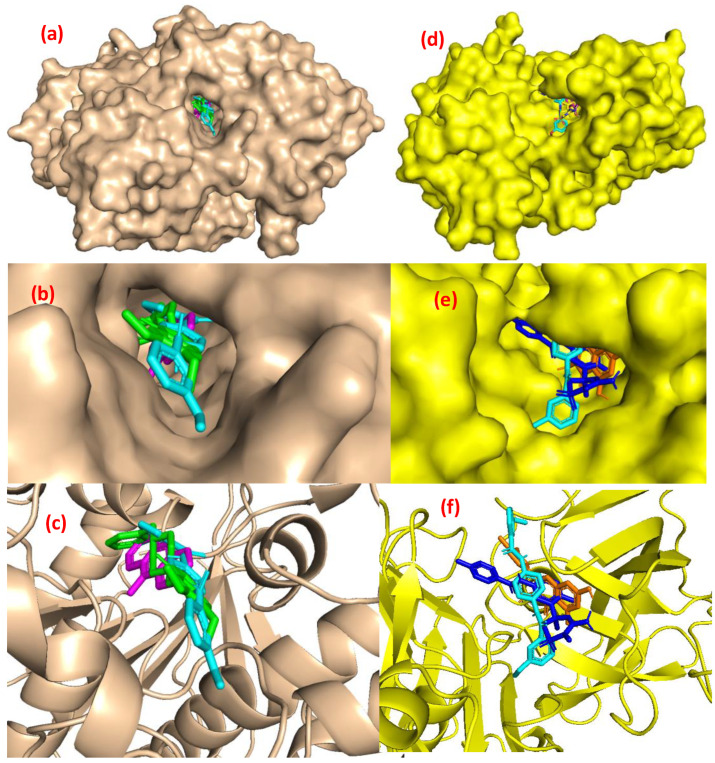
Structures of complexes: AChE (**a**–**c**) with donepezil (green), tacrine (magenta), **JW8** (cyan), and BACE1 (**d**–**f**) with quercetin (orange), verubecestat (blue), and **JW8** (cyan).

The active sites of both enzymes with superimposed docked ligands (donepezil, tacrine, **JW8** for AChE and quercetin, verubecestat, and **JW8** for BACE1) are shown in Figure 4.

**Figure 5:**
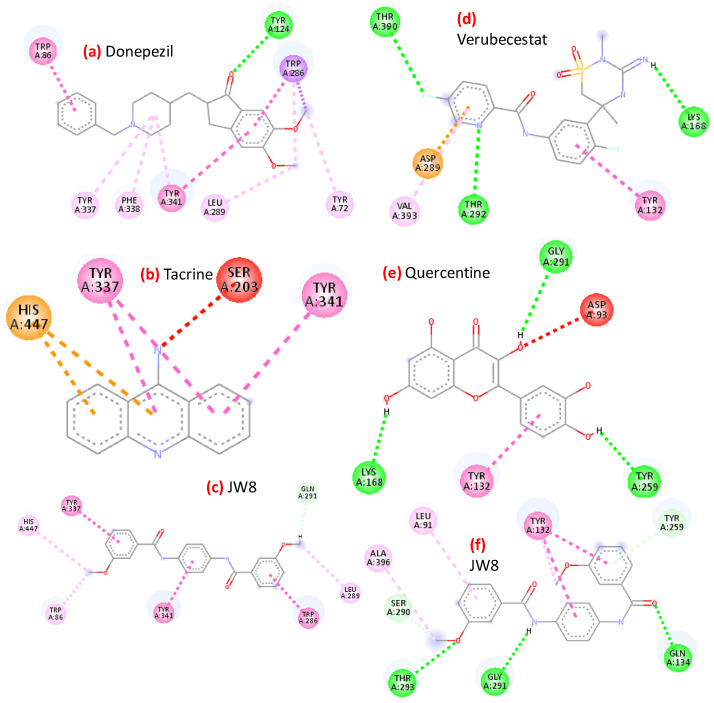
Interactions between (1) AChE (**a**–**c**) and (2) BACE1 (**d**–**f**) and the best inhibitors resulting from docking studies.

The positions of individual ligands do not differ strongly for AChE; instead, they almost overlap. For BACE, a clear difference in ligand locations was apparent, with **JW8** entering one of the rings in a cavity where no other ligands were located (see Figure 4e). On the other hand, the relative locations of both centers noticeably varied. While the active site of AChE is a cavity buried deeply in the structure of the enzyme, for BACE1, it is located much closer to the surface, in a gorge at the edge of the protein. This has important implications for patterns of interaction when stabilizing the complex, as shown in Figure 5.

For AChE, nonpolar interactions mainly involve pi electrons, which are somewhat weaker than polar hydrogen bonds. Tacrine interactions clearly overlap with those for **JW8**, as indicated by two pi–pi-type contacts with Tyrosine. The arrangement of these bonds coincided with the docking poses, with one of the terminal rings of tacrine positioned where the internal ring of **JW8** was located. In turn, the inner ring of tacrine overlapped with one of the two outer rings of **JW8**. Corresponding analogies could also be noted when comparing the interactions of **JW8** and donepezil. Here, too, there was contact with Tyrosine-341 (TYR-341) shared by three ligands. The interactions of the tested inhibitors with BACE1 were somewhat different—more hydrogen bonds appeared, both classical and weak. Again, a common interaction for all inhibitors is an interaction with tyrosine (π–π with TYR-132). Nevertheless, there is no other common contact related to the aforementioned differences in docking poses. Accordingly, it is concluded that the differences between the poses of individual inhibitors are greater for BACE1 than for AChE.

### 2.4. Molecular Dynamics

**Figure 6:**
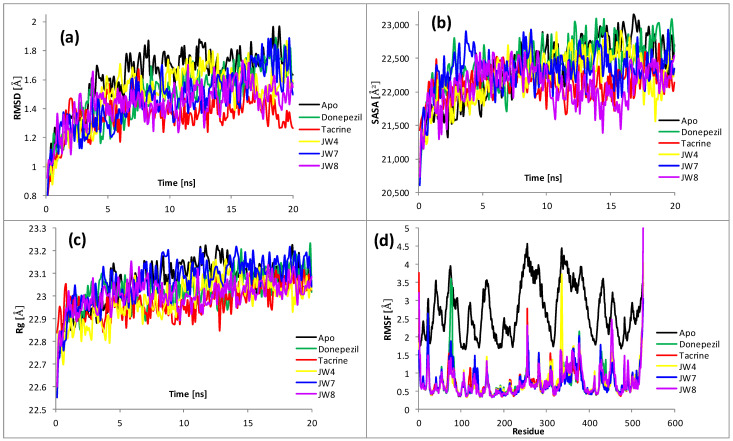
Analysis of RMSD, SASA, Rg, and RMSF of unliganded acetylcholinesterase (AChE Apo) and five complexes during 20 ns of MD simulations: (**a**) root mean square deviation (RMSD) for Cα atoms; (**b**) solvent accessible surface area (SASA); (**c**) radius of gyration (Rg); (**d**) root mean square fluctuation (RMSF) values for each residue averaged over the entire simulation.

**Figure 7:**
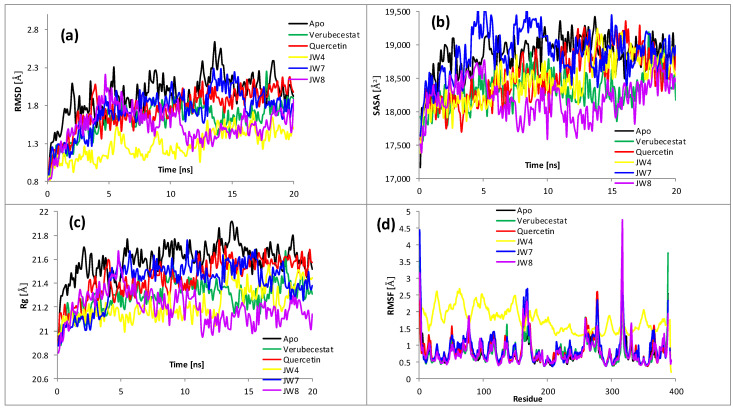
Analysis of RMSD, SASA, Rg, and RMSF of unliganded β-secretase (BACE1 Apo) and five complexes during 20 ns of MD simulations: (**a**) root mean square deviation (RMSD) for Cα atoms; (**b**) solvent accessible surface area (SASA); (**c**) radius of gyration (Rg); (**d**) root mean square fluctuation (RMSF) values for each residue averaged over the entire simulation.

To proceed beyond the static image yielded by the docking, molecular dynamic simulations were performed. Complexes with the most promising derivatives (i.e., **JW4**, **JW7**, and **JW8**) and reference drugs (donepezil, tacrine, verubecestat, and quercetin) were selected for modeling. The 20 ns simulations were launched, starting from the best docking poses. The values of basic descriptive statistics (arithmetic means, standard deviations (SDs), and coefficients of variation (h = SD/arithmetic mean)) for all complexes are given in the Supporting Information (Table S1 for AChE and Table S2 for BACE1). These statistics, together with a thorough analysis of the resulting trajectories (Figure 6 and Figure 7), help to draw more accurate conclusions regarding the underlying mechanism of inhibition. For both enzymes, the RMSD values (Figure 6a and Figure 7a) were rather small (i.e., <2 Å) for all tested systems and did not increase during the period of simulation, indicating the stability of all forms, which is consistent with results reported in [7]. For AChE, a comparison of the averaged values (1.62, 1.49, 1.36, 1.50, 1.46, and 1.44 Å for apo-form, donepezil, tacrine, **JW4**, **JW7**, and **JW8**, respectively) revealed the slight stabilizing effect of the ligands. Again, the results from ref. [7] also suggest a slight increase in the stability of the complexes, albeit with a different type of ligand. This effect was significant for tacrine, although it was minor and similar for other ligands, and the differences with respect to the unliganded form were noticeable for all complexes. The same is true for BACE1, where complexes also appeared to be more stable than the apo-form. Here, the averages were 1.94, 1.62, 1.76, 1.28, 1.74, and 1.55 Å for the apo-form, verubecestat, quercetin, **JW4**, **JW7**, and **JW8**, respectively. It is interesting to observe that the most pronounced stabilizing effect was exerted by **JW4** for both enzymes. However, an appreciable difference could also be observed here; for AChE, the RMSD of the complex with **JW4** reached its maximum around 12–13 ns and then monotonically descended, while for BACE1, it was difficult to distinguish such a clear maximum. A comparison of the RMSD values averaged over all ligands (1.45 Å for AChE and 1.59 Å for BACE1) and their standard deviations (0.17 Å for AChE and 0.23 Å for BACE1) illustrates the dependence of RMSD variability on the enzyme. Generally, AChE exhibits a higher stability during modeling, although this difference is not very significant.

A higher SASA value indicates an increase in the protein volume; thus, one may expect to have a low fluctuation during simulation. The binding of any small moiety can significantly influence the protein structure, thus altering SASA. This can be examined by inspecting SD values, which are a measure of fluctuations. For AChE, SD averaged over complexes of 310 Å^2^ was significantly lower than the corresponding value for the ligand-free form (460 Å^2^). All ligands significantly reduced this parameter, with tacrine decreasing the most. In the case of BACE1, only verubecestat and **JW8** reduced SD, and the value averaged over all ligands was also smaller than that for the apo-form. This meant that the considered inhibitors significantly stabilized acetylcholinesterase, but β-secretase was weaker than all of them. The exception was **JW8**, which reduced the expansions of both enzymes while clearly having a stronger effect on AChE. As can be seen from Tables S1 and S2, the average values of SASA were noticeably lower for BACE1, which is easy to explain since BACE1 consists of fewer nonhydrogen atoms. However, the standard deviations and coefficients of variation were noticeably larger for BACE1, thus supporting the claim of noteworthy fluctuations in SASA and indicating relatively less stability.

The radius of gyration (Rg) is a measure of the compactness of the structure. The lower degree of fluctuation and its steadiness during the simulation means that the system is more compact and rigid. For both unliganded enzymes and complexes, it exhibits small deviations (fraction of Å), indicating that the systems are tightly packed without significant structural changes (Figure 6c and Figure 7c, Tables S1 and S2). The average Rgs for BACE1 is slightly lower than for AChE, which may be related to the smaller size of β-secretase. For AChE, all complexes were observed to slightly decrease Rg when compared to the apo-form for almost the entire simulation. **JW7**, with an average value of only a few hundredths Å larger than that for the apo-form, is an exception. In general, the influence of the ligands is minor for both enzymes; the average of the complexes differs from apo-forms by a maximum of 0.04 Å. The averaged standard deviations and coefficients of variation are slightly larger for BACE1, supporting the assertion that this enzyme and its complexes are less stable. As for SASA, **JW8** affects the Rg of both proteins most noticeably, making them more compact and rigid. However, this influence is not very pronounced.

The root mean square fluctuation (RMSF) gauges the average deviation of a protein residue over time from the reference point. This analysis is helpful in understanding the way in which the flexibility of specific parts of the macromolecule is altered by bound inhibitors. The results are plotted in Figure 6d (AChE) and Figure 7d (BACE1). For AChE, the RMSF for the unliganded form was significantly higher than that of complexes for almost all residues. Only single RMSF values for these complexes were close to those for the apo-form. Their averages were about 2.74 Å for the apo-form and 0.74–0.77 Å for the complexes, thus indicating the substantial effect of all ligands on the flexibility of the enzyme. This can be considered as contributing to explaining the mechanism of inhibition—an increase in the stiffness of the enzyme simultaneously causes a decrease in its activity. The situation is somewhat different for BACE1, where the largest RMSF shows a complex with **JW4**. The averaged fluctuations over a 20 ns period are similar for both proteins. It is worth noting, however, that the highest mobility of a single residue exhibits **JW8** (specifically LIS-317); in addition, for **JW7** and verubecestat, some residues show higher mobility than any from **JW4**.

The ability to form polar interactions for the tested inhibitors was examined by detecting the number of hydrogen bonds between the receptor and ligand during 20 ns of the simulation. The results are shown in Table 2 (AChE) and Table 3 (BACE1). The data show that dynamic interactions were not fundamentally different from the static ones in docking. The dominant interactions were with tyrosine, specifically with TYR-337 (for all ligands) and TYR-124, which occurred for all ligands except **JW8**. Hydrogen bonds with HIS-447 and SER-203, already present at the docked structure, were also detected. Thus, it may be concluded that the position of ligands relative to the receptor did not change significantly over time. This result could be expected from the analysis of the structure of the AChE active center, which has a form of cavity deeply “buried” in the structure of the enzyme (see Figure 4). It is clear that obtaining the ligand from there is problematic, if at all possible. However, within this cavity, various arrangements of ligands are likely. As can be seen from Table 2, the alignment of **JW8** is slightly different from the other inhibitors, including the reference substances (donepezil and tacrine). The most common interactions for **JW8** (with phenylalanine PHE-295 and PHE-338) are absent or marginal compared with the other ligands, which could suggest a different mechanism of enzyme inhibition by **JW8**. Since the active center of AChE is a cavity whereby the ligand is “trapped”, access to it is mechanically blocked. This supports the hypothesis of the competitive inhibition mode. Differences in the trajectories of individual ligands are associated with different variants of the same mode of inhibition. When discussing the pattern of interactions for BACE1, the differences between these binding sites of the two enzymes must be taken into account. The active center of BACE1 resembles a gorge on the surface rather than a cavity. Therefore, the freedom of movement of the ligand is far superior to AChE. As can be seen from Table 3, complexes with **JW8** and verubecestat show substantially fewer polar interactions than the remaining ones. Visual inspection of the appropriate trajectories indicates that both these ligands detach from the bottom of the gorge and relocate on the surface next to the binding site, which could impair the enzyme’s functionality. This suggests a noncompetitive rather than competitive inhibition mode.

### 2.5. QSAR

Another analysis focused on finding values for pharmacokinetic parameters that might be important for assessing the suitability of molecules as drug candidates. Combined with the previous consideration of the exact mechanism of interaction of the tested compounds with enzymes, this could help compare the bioactivity of the proposed derivatives in relation to the reference compounds, thus allowing for a comprehensive estimation of the suitability of the substance for further experiments. More than twenty pharmacokinetic parameters were evaluated, in particular, those related to toxicity, properties in relation to the glycoprotein, absorption in the body, penetration across the blood–brain barrier, and general criteria for assessing the suitability of a molecule as a drug. The results are summarized in Table 3, and they suggest the low toxicity of the proposed inhibitors and their proper absorption in the body. Of particular interest are the pre-logP values, which fall into two groups. The new derivatives and donepezil are much more lipophilic than the other reference substances. The results suggest the low toxicity of the proposed inhibitors and their proper absorption into the body. Low values of the Sa-score indicate the relative ease of synthesis. The estimated logP values fall into two groups; the new derivatives and donepezil are much more lipophilic than the other reference substances. Our proposed drug candidates also meet the general requirements for drugs, which are no worse than the reference drugs already on the market, where the drug-likeness score is highest for berubecestat, **JW4**, and **JW8**. It is interesting to note that **JW8** is the only one of the molecules considered here that meets all the four rules of thumb to assess drug-likeness.

## 3. Materials and Methods

### 3.1. Chemistry

#### 3.1.1. General Information

Thin layer chromatography experiments (TLC) were carried out on silica gel (Merck; 60 Å F254). Spots were located with UV light (254 and 366 nm), and 1H-NMR and 13C-NMR spectra were recorded on a Bruker AC 400F spectrometer (Bruker Corp., Fällanden, Switzerland) using TMS as the internal standard; chemical shifts are reported in ppm. Chemical shifts (ppm) were relative to TMS and used as an internal standard. Multiplicities are marked as s = singlet, d = doublet, t = triplet, q = quartet, qu = quintet, and m = multiplet. ESI-HRMS spectra were obtained on an Agilent 6530 Accurate-Mass Q-TOF ESI and LC/MS system (Agilent Poroshell 120 EC-C18 2.7 microm 3.0 × 150 mm, flow 0.4 mL/min, A: water, B: MeOH. 0 60% A, 2.00 5% A, 6.00 5% A). The spectra of the new derivatives reported here have been placed in the Supplementary Materials.

#### 3.1.2. Synthesis and Spectroscopic Analysis of Benzamides

- N,N′-(1,3-phenylene)bis(3,4,5-trimethoxybenzamide) **JW1**

Starting materials: benzene-1,3-diamine (0.174 g; 1.61 mmol), DIPEA 0.41 cm^3^, 3,4,5-trimethoxybenzoyl chloride (0.744 g, 3.22 mmol). ^1^H NMR (d_6_-DMSO): 3.74 (s, 3H, OCH_3_), 3.58 (s, 6H, OCH_3_), 7.30 (s, 4H, Ar-H), 7.34 (dd, 2H, Ar-H), 7.49 (t, 1H, Ar-H), (8.21 (s, 1H, Ar-H), 10.18 (s, 2H, CONH); ^13^C NMR (d_6_-DMSO): 56.10 (2OCH_3_), 60.13 (OCH_3_), 105.30 (4CH), 113.55 (CH), 116.44 (2CH), 128.59 (2C), 129.97 (CH), 139.22 (2C), 140.30 (2C), 152.63 (4C), 164.84 (2CONH); yield: 72.81%; m.p. 175–177 °C; M = 496.52 g/mol; HRMS (ESI): [M+H]^+^ 497.1928, calculated for C_26_H_29_N_2_O_8_^+^ 497.1918

- N,N′-(1,3-phenylene)bis(3,5-dimethoxybenzamide) **JW2**

Starting materials: benzene-1,3-diamine (0.174 g; 1.61 mmol), DIPEA 0.41 cm^3^, 3,5-dimethoxybenzoyl chloride (0.646 g, 3.22 mmol). ^1^H NMR (d_6_-DMSO): 3.83 (s, 6H, OCH_3_), 6.72 (s, 2H, Ar-H), 7.14 (s, 4H, Ar-H), 7.32 (t, 1H, Ar-H), 7.49 (dd, 2H, Ar-H), 8.28 (s, 1H, Ar-H), 10.23 (s, 2H, CONH); ^13^C NMR (d_6_-DMSO): 55.51 (2OCH_3_), 103.36 (2CH), 105.67 (2CH), 113.32 (CH), 116.36 (2CH), 128.59 (CH), 128.57 (CH), 136.95 (2C), 139.20 (2C), 160.38 (4C), 164.84 (2CONH); yield: 84.96%; m.p. 188–190 °C; M = 436.46 g/mol; HRMS (ESI): [M+H]^+^ 437.1714, calculated for C_24_H_25_N_2_O_6_^+^ 437.1707.

- N,N′-(1,3-phenylene)bis(3,4-dimethoxybenzamide) **JW3**

Starting materials: benzene-1,3-diamine (0.174 g; 1.61 mmol), DIPEA 0.41 cm^3^, 3,4-dimethoxybenzoyl chloride (0.646 g, 3.22 mmol). ^1^H NMR (d_6_-DMSO): 3.84 (s, 3H, OCH_3_), 3.86 (s, 3H, OCH_3_), 7.09 (dd, 2H, Ar-H),), 7.31 (t, 2H, Ar-H), 7.48 (t, 1H, Ar-H), 7.57 (s, 2H, A-H), 7.66 (dd, 2H, Ar-H), 8.26 (s, 1H, Ar-H), 10.12 (s, 2H, CONH); ^13^C NMR (d_6_-DMSO): 55.64 (OCH_3_), 55.68 (OCH_3_), 112.95 (2CH), 113.13 (CH), 116.22 (CH), 117.29 (2CH), 128.57 (2C), 129.54 (CH), 136.35 (2C), 139.20 (2C), 159.17 (2C), 164.84 (2CONH); yield: 77.76%; m.p. 128–130 °C; M = 436.46 g/mol; HRMS (ESI): [M+H]^+^ 437.1713, calculated for C_24_H_25_N_2_O_6_^+^ 437.1707.

- N,N′-(1,3-phenylene)bis(3-methoxybenzamide) **JW4**

Starting materials: benzene-1,3-diamine (0.174 g; 1.61 mmol), DIPEA 0.41 cm^3^, 3-methoxybenzoyl chloride (0.549 g, 3.22 mmol). ^1^H NMR (d_6_-DMSO): 3.85 (s, 3H, OCH_3_), 7.15 (s, 1H, Ar-H), 7.17 (s, 1H, Ar-H), 7.31 (t, 2H, Ar-H), 7.45 (t, 1H, Ar-H), 7.45 (dd, 4H, Ar-H), 7.51 (d, 2H, A-H), 7.57 (dd, 2H, Ar-H), 8.32 (s, 1H, Ar-H), 10.28 (s, 2H, CONH); ^13^C NMR (d_6_-DMSO): 55.34 (OCH_3_), 112.95 (2CH), 113.13 (2CH), 116.22 (CH), 117.29 (2CH), 119.94 (2CH), 128.57 (2CH), 129.54 (CH), 136.35 (2C), 139.28 (2C), 159.17 (2C), 165.24 (2CONH); yield: 85.00%; m.p. 171–173 °C; M = 376.41 g/mol; HRMS (ESI): [M+H]^+^ 377.1503, calculated for C_22_H_21_N_2_O_4_^+^ 377.1496.

- N,N′-(1,4-phenylene)bis(3,4,5-trimethoxybenzamide) **JW5**

Starting materials: benzene-1,4-diamine (0.174 g; 1.61 mmol), DIPEA 0.41 cm^3^, 3,4,5-trimethoxybenzoyl chloride (0.744 g, 3.22 mmol). ^1^H NMR (d_6_-DMSO): 3.74 (s, 3H, OCH_3_), 3.88 (s, 6H, OCH_3_), 7.30 (s, 4H, Ar-H), 7.73 (s, 4H, Ar-H), 10.14 (s, 2H, CONH); ^13^C NMR (d_6_-DMSO): 56.11 (2OCH_3_), 60.13 (OCH_3_), 105.23 (4CH), 120.96 (4CH), 130.07 (2C), 134.88 (2C), 140.23 (2C), 152.66 (4C), 164.69 (2CONH); yield: 85.00%; m.p. > 200 °C; M = 496.52 g/mol; HRMS (ESI): [M+H]^+^ 497.1927, calculated for C_26_H_29_N_2_O_8_^+^ 497.1918.

- N,N′-(1,4-phenylene)bis(3,5-dimethoxybenzamide) **JW6**

Starting materials: benzene-1,4-diamine (0.174 g; 1.61 mmol), DIPEA 0.41 cm^3^, 3,5-dimethoxybenzoyl chloride (0.646 g, 3.22 mmol). ^1^H NMR (d_6_-DMSO): 3.83 (s, 6H, OCH_3_), 6.55 (s, 2H, Ar-H), 7.10 (s, 4H, Ar-H), 7.74 (s, 4H, Ar-H), 10.18 (s, 2H, CONH); ^13^C NMR (d_6_-DMSO): 55.51 (2OCH_3_), 103.31 (2CH), 105.59 (4CH), 120.80 (4CH), 134.89 (2C), 137.02 (2C), 160.39 (4C), 164.84 (2CONH); yield: 74.50%; m.p. > 200 °C; M = 436.46 g/mol; HRMS (ESI): [M+H]^+^ 437.1713, calculated for C_24_H_25_N_2_O_6_^+^ 437.1707.

- N,N′-(1,4-phenylene)bis(3,4-dimethoxybenzamide) **JW7**

Starting materials: benzene-1,4-diamine (0.174 g; 1.61 mmol), DIPEA 0.41 cm^3^, 3,4-dimethoxybenzoyl chloride (0.646 g, 3.22 mmol). ^1^H NMR (d_6_-DMSO): 3.84 (s, 3H, OCH_3_), 3.85 (s, 3H, OCH_3_), 7.08 (d, 2H, Ar-H), 7.55 (d, 2H, Ar-H), 7.62 (dd, 2H, Ar-H), 7.73 (s, 4H, Ar-H), 10.09 (s, 2H, CONH); ^13^C NMR (d_6_-DMSO): 55.64 (2OCH_3_), 55.64 (2OCH_3_), 110.92 (2CH), 111.00 (2CH), 120.77 (2CH), 120.98 (4CH), 127.04 (2C),134.49 (2C), 148.30 (2C), 151.57 (2C), 164.70 (2CONH); yield: 71.88%; m.p. > 200 °C; M = 496.52 g/mol; HRMS (ESI): [M+H]^+^ 437.1716, calculated for C_24_H_25_N_2_O_6_^+^ 437.1707.

- N,N′-(1,4-phenylene)bis(3-methoxybenzamide) **JW8**

Starting materials: benzene-1,3-diamine (0.174 g; 1.61 mmol), DIPEA 0.41 cm^3^, 3-methoxybenzoyl chloride (0.549 g, 3.22 mmol). ^1^H NMR (d_6_-DMSO): 3.85 (s, 3H, OCH_3_), 7.15 (d, 2H, Ar-H), 7.44 (t, 2H, Ar-H), 7.45 (d, 2H, Ar-H), 7.46 (d, 2H, Ar-H), 7.55 (s, 4H, Ar-H), 10.22 (s, 2H, CONH); ^13^C NMR (d_6_-DMSO): 55.34 (OCH_3_), 112.87 (2CH), 117.25 (2CH), 119.84 (2CH), 120.72 (4CH), 129.55 (2CH),134.93 (2C), 136.39 (2C), 159.19 (2C), 165.30 (2CONH); yield: 78.70%; m.p. > 200 °C; M = 376.41 g/mol; HRMS (ESI): [M+H]^+^ 377.1503, calculated for C_22_H_21_N_2_O_4_^+^ 377.1496.

- 3,4,5-trimethoxy-N-(3-nitrophenyl)benzamide **MB1**

Starting materials: 3-nitroaniline (0.33 g; 2.38 mmol), DIPEA 0.46 cm^3^, 3,4,5-trimethoxybenzoyl chloride (0.55 g, 2.38 mmol). ^1^H NMR (d_6_-DMSO): 3.75 (s, 3H, OCH_3_), 3.88 (s, 6H, OCH_3_), 7.32 (s, 2H, Ar-H), 7.65–7.67 (t, 1H, Ar-H), 7.96 (d, 1H, Ar-H), 7.98 (d, 1H, Ar-H), 8.74 (s, 1H, Ar-H), 10.54 (s, 1H, CONH)); ^13^C NMR (d_6_-DMSO): 56.16 (2OCH_3_), 60.17 (OCH_3_), 105.45 (2CH), 114.54 (CH), 118.17 (CH), 126.35 (CH), 129.26 (C), 130.09 (CH), 140.29 (C), 140.69 (C), 147.90 (C), 152.70 (2C), 165.38 (CONH); yield: 77.41%; m.p. 180–182 °C; M = 332.31 g/mol; HRMS (ESI): [M+H]^+^ 333.1087, calculated for C_16_H_17_N_2_O_6_^+^ 333.1081.

- 3,4,5-trimethoxy-N-(3,4,5-trimethoxyphenyl)benzamide **MB3**

Starting materials: 3,4,5-trimethoxyaniline (0.39 g; 2.13 mmol), DIPEA 0.41 cm^3^, 3,4,5-trimethoxybenzoyl chloride (0.49 g, 2.13 mmol). ^1^H NMR (d_6_-DMSO): 3.65 (s, 3H, OCH_3_), 3.74 (s, 3H, OCH_3_), 3.78 (s, 6H, OCH_3_), 3.88 (s, 6H, OCH_3_), 7.18 (s, 2H, Ar-H), 7.28 (s, 2H, Ar-H), 10.05 (s, 1H, CONH); ^13^C NMR (d_6_-DMSO55.76 (2OCH_3_), 56.14 (2OCH_3_), 60.14 (2OCH_3_), 98.31 (2CH), 105.26 (2CH), 105.67 (2CH), 129.99 (C), 133.77 (C), 135.18 (C), 140.32 (C), 152.63 (4C), 164.71 (CONH); yield: 84.25%; m.p. > 200 °C; M = 377.39 g/mol; HRMS (ESI): [M+H]^+^ 378.1554, calculated for C_19_H_24_NO_7_^+^ 378.1547.

- 3,4,5-trimethoxy-N-(3,4,5-trimethoxybenzyl)benzamide **MB4**

Starting materials: 3,4,5-trimethoxybenzylaniline (0.3469 mL; 2.04 mmol), DIPEA 0.41 cm^3^, 3,4,5-trimethoxybenzoyl chloride (0.47 g, 2.04 mmol). ^1^H NMR (d_6_-DMSO): 3.64 (s, 3H, OCH_3_), 3.71 (s, 3H, OCH_3_), 3.75 (s, 6H, OCH_3_), 3.83 (s, 6H, OCH_3_), 4.42–4.44 (d, 2H, CH_2_), 6.66 (s, 2H, Ar-H), 7.225 (s, 2H, Ar-H), 8.92–8.95 (t, 1H, CONH); ^13^C NMR (d_6_-DMSO): 43.02 (CH_2_), 55.83 (OCH_3_), 55.98 (OCH_3_), 59.99 (OCH_3_), 60.06 (OCH_3_), 104.74 (2CH), 104.84 (2CH), 129.58 (C), 135.37 (C), 136.42 (C), 139.95 (C), 152.61 (2C), 152.84 (2C), 164.71 (CONH); yield: 72.06%; m.p. 177–179 °C; M = 391.42 g/mol; HRMS (ESI): [M+H]^+^ 392.1712, calculated for C_20_H_26_NO_7_^+^ 392.1704.

### 3.2. Biological Activity

#### 3.2.1. In Vitro Inhibition Studies on AChE

The spectroscopic method we used was previously described in detail [26]. This method involved the use of an acetylcholinesterase inhibitor screening kit (catalog number MAK324). This kit, as well as the enzyme (purified AChE—catalog number C3389) and donepezil, were purchased from Sigma-Aldrich. Enzyme solutions were prepared according to the instructions in double-distilled water. The solutions of the compounds in DMSO were diluted with a 0.1 M phosphate buffer KH_2_PO_4_/K_2_HPO_4_ (pH 7.5) at room temperature to obtain solutions of 1–100 mM. An Infinite M200 fluorescence spectrophotometer (TECAN, Männedorf, Switzerland) was used to read absorbance (ex. 412 nm). Tests were performed on a transparent 96-well plate with a flat bottom. The final concentrations of donepezil and test compounds were 1, 10, 20, 50, and 100 mM. The absorbance of each sample was measured at 412 nm after 0 min and after 10 min. The calculated percentage of inhibition correlated with acetylcholinesterase activity. From the obtained data, the concentration of the compound that caused a 50% decrease in activity, i.e., the IC_50_ value (µM), was calculated. The experiment was repeated three times.

#### 3.2.2. In Vitro Inhibition Studies on β-Secretase (BACE1)

Also, a β-secretase (BACE1) activity assay kit was purchased from Sigma-Aldrich (catalog number CS0010) and was used as described previously [26]. In this experiment, an enhancement of the fluorescence signal after substrate cleavage via BACE1 was observed [27]. The concentrations of compounds were the same as in the assay with AChE. The assay was performed using a black 96-well microplate. Fluorescence was measured on an Infinite M200 fluorescence spectrophotometer (TECAN, Männedorf, Switzerland) (ex. 320 nm; em. 405 nm) in triplicate experiments with a negative control (no enzyme) and a positive control (enzyme activity supplied). Secretase activity correlated with fluorescence value. Its 50% decrease was calculated as IC_50_ (µM).

### 3.3. Molecular Docking

**Figure 8:**
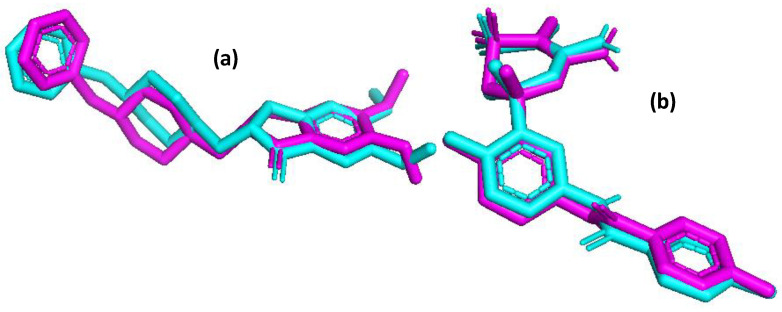
The alignment of the native ligands donepezil PDB:E20 (**a**) and verubecestat PDB:66F (**b**) (cyan) compared with the best poses from the redocking procedures (magenta).

To gain a better understanding of the activity of new species, a molecular docking study was carried out using the latest (1.2.5) version of AutoDock Vina software [28,29]. Pymol [30] and BIOVIA Discovery Studio Visualizer [31] programs were used to visualize the results. The crystal structures of human AChE (PDB:7E3H, resolution 2.45 Å [32]) and BACE1 (PDB:5HU1, resolution 1.50 Å [33]) were downloaded from the Protein Data Bank. The selection of these structures was motivated by the embedding of drug ligands, namely donepezil (PDB: E20) in 7E3H and verubecestat (PDB:66F) in 5HU1; the docking process was carried out for the A subunits of both proteins. Both enzymes were pre-prepared for docking by purifying their structures from solvent molecules and other co-crystallized individuals, adding polar H atoms and Kollman charges [34]. The cubical search box (20 × 20 × 20 Å) was set to cover the ligands during the docking procedure. To improve its accuracy, the EXHAUSTIVENESS was set to 100 (default = 8), guaranteeing the best performance [35]. A redocking process was carried out to validate the results. The poses with the best docking score were compared with the scores of E20 (donepezil) and 66F (verubecestat) from crystal structures. Both conformations were found to be in satisfactory agreement, with RMSD = 1.175 Å for donepezil and 1.041 Å for verubecestat. The aligned ligands are visualized in Figure 8 below

### 3.4. Molecular Dynamics

Molecular dynamic studies were accomplished with NAMD 2.14 [36]/VMD [37] software leveraged by the CHARMM22 force field [38] with CMAP correction to proteins [39]. The individual parameter files (.prm) for the ligands were prepared with the Ligand Reader and Modeler online tool from the CHARMM-GUI online environment [40,41]. Simulations were conducted with explicit water molecules in a periodically repeating cube with a margin of 5 Å. The protein–ligand complexes were solvated and ionized with the tools provided by the VMD program. The ionized systems were minimized with 10,000 integration steps, gradually heated from 0 K to 310 K with 2 K increments, and, finally, equilibrated with an additional 50,000 integration steps of 2 fs. An unrestrained production run of 20 ns where a single step = 2 fs was then performed for subsequential analysis and frames were saved for every 2000 steps. Constant pressure (p = 1 atm) was imposed in the Langevin piston methodology with a decay period of 100 fs. A constant temperature of 310 K was enforced by Langevin dynamics with a damping coefficient of 5 (1/ps). The trajectory properties were calculated with scripts provided by the NAMD/VMD developer. In particular, the following parameters were investigated: the root mean square deviation (RMSD), solvent-accessible surface area (SASA), the radius of gyration (Rg), the root mean square fluctuation (RMSF), and H-bond patterns.

### 3.5. QSAR Modeling

All values from Table 3 were obtained with the online tool Admet SAR [42].

## 4. Conclusions

The substituted benzamides are considered to be bioactive compounds and can be treated as therapeutic material. It can be concluded that this class of compounds certainly holds great promise toward good active leads in medicinal chemistry [8]. In the present paper, different benzene substitutions and amounts of methoxy groups were selected to identify possible structure–activity relationships. 

The results of molecular docking show the three most promising derivatives (**JW4**, **JW7**, and **JW8**), which are in accordance with IC_50_ measurements. Quantitative results suggesting the **JW8** derivative as the most promising are not substantially different for new derivatives and reference compounds. The active sites of these two enzymes are obviously dissimilar for AChE is in the form of a cavity, while BACE1 is a gorge located almost on the surface of the enzyme, which suggests different mechanisms of inhibition for both enzymes. Molecular dynamic simulations lead to the conclusion that the arrangement of individual ligands for acetylcholinesterase is similar and does not fundamentally change over time. All ligands remain in the cavity throughout the simulation period, suggesting a competitive inhibition mode. The main polar contact is with TYR-337, except for **JW8**, for which the hydrogen bonding scheme was noticeably altered. The results are different for β-secretase, where **JW8** and verubecestat behave differently from the rest; they migrate from the active center to embed in its immediate vicinity, which tends to suggest a noncompetitive inhibition mode. The molecular dynamic simulations also revealed that all ligands had a small and similar effect on the stabilization of both enzymes, where, more notably, there was a decrease in enzyme flexibilities. Increased protein stiffness can also hamper enzyme activity. The suitability of new derivatives as drug candidates is also confirmed with QSAR calculations, which suggest their low toxicity and good bioavailability.

As computer calculations revealed, the values of the docking score and ligand efficiency were the same for **JW8** and **JW4**. However, this difference was noticeable in the results of an in vitro study. The only difference in structure was the positioning of substituents in the central benzene ring. The positioning of the meta–meta was found to be less favorable, and we observed this in both activities toward AChE and BACE1. From these results, we also inferred that the presence of one methoxy group was sufficient to obtain good activity, as we noted that additional methoxy groups in the structure of the compounds **JW5**, **JW6**, and **JW7** reduced the inhibitory activity against AChE and BACE1. This may suggest that there is a spatial hindrance to the binding of these compounds to the active sites of the enzymes. Therefore, **JW8** may be considered a potential lead compound for the development of new anti-Alzheimer drugs in the future.

In summary, the results obtained confirm our assumptions for the synthesis of multi-target compounds: inhibitory activity against enzymes with different action profiles. The results of the in vitro research were also confirmed with molecular docking studies, leading us to the conclusion that these structures might have an interesting future as a template with which to develop new analogs with potential anti-neurodegenerative properties.

## Figures and Tables

**Table 1:** In vitro inhibition of AChE and BACE1 of the new and reference compounds.

Compound	AChE IC_50_ (µM) ^a^			BACE1 IC_50_ (µM) ^b^		
	IC_50_ (µM)	Docking Score (kcal/mol)	Ligand Efficiency (kcal/mol*atom)	IC_50_ (µM)	Docking Score (kcal/mol)	Ligand Efficiency (kcal/mol*atom)
**JW1**	0.77 ± 0.018	−9.8	0.27	54.44 ± 1.30	−7.6	0.21
**JW2**	2.57 ± 0.063	−10.3	0.32	73.98 ± 1.61	−8.0	0.25
**JW3**	0.41 ± 0.028	−10.4	0.32	40.16 ± 8.12	−7.7	0.24
**JW4**	1.20 ± 0.013	−11.2	0.40	81.37 ± 4.11	−8.2	0.29
**JW5**	0.068 ± 0.058	−10.0	0.28	9.29 ± 3.02	−7.3	0.20
**JW6**	0.19 ± 0.033	−10.7	0.33	16.66 ± 0.25	−8.0	0.25
**JW7**	0.07 ± 0.003	−10.8	0.34	12.76 ± 0.23	−8.1	0.25
**JW8**	0.056 ± 0.043	−11.2	0.40	9.01 ± 0.28	−8.1	0.29
**MB1**	0.67 ± 0.008	−9.4	0.39	40.45 ± 2.20	−7.0	0.29
**MB3**	0.11 ± 0.078	−8.8	0.32	20.22 ± 5.13	−6.4	0.27
**MB4**	1.17 ± 0.015	−8.7	0.31	87.67 ± 6.22	−6.8	0.25
donepezil	0.046 ± 0.013	−11.6	0.41	- ^c^	−8.7	0.31
tacrine	0.274 ± 0.08	−8.9	0.59	- ^c^	−6.6	0.23
quercetin ^d^	- ^c^	−9.3	0.42	4.89 ± 2.31	−8.4	0.56
verubecestat					−8.9	0.40

^a^ AChE from Electrophorus electricus (electric eel); IC_50_, inhibitor concentration (mean ± SD of three independent experiments) resulting in 50% inhibition of AChE; ^b^ BACE1 from equine serum; IC_50_, inhibitor concentration (mean ± SD of three independent experiments) resulting in 50% inhibition of BACE1; ^c^ n.d., not determined; ^d^ quercetin was used as a standard positive control agent.

**Table 2:** List of H bonds between the proposed inhibitors and residues in the active site of AChE. The interaction type indicates whether the ligand is a donor (D) or an acceptor (A). Only residues with an occupancy of no less than 2% are shown.

Ligand	AChE			Ligand	BACE1		
	Residue	Type	Occupancy (%)		Residue	Type	Occupancy (%)
Donepezil	TYR-124	A	62.5	Verubecestat	GLN-134	A	7.5
	TYR-125	D	12.5		LYS-168	A/D	5
	TYR-337	A	9.5		LEU-91	A	4.5
	TRP-286	A	9		THR-94	A	2
	TYR-341	D	6.5		GLY-291	A	2
	PHE-295	A	4.5	Quercetin	ASP-289	D	54
	SER-293	D	2		SER-290	A	7
	TYR-72	D	2		THR-94	A	5.5
	SER-203	D	2		THR-390	A	4.5
Tacrine	TYR-124	A	62.3		TYR-259	A	4.5
	TYR-337	A	9.5		LYS-285	A	2.5
	SER-203	D	3.5	**JW4**	ASP-93	D	53.5
	HIS-447	D	2		GLY-291	D	50
**JW4**	TYR-337	A	59		ILE-171	A	2.5
	TYR-124	D	33		THR-94	D	3
	HIS-447	D	19		THR-293	D	2
	TYR-124	A	8.5	**JW7**	TRP-137	A	47
	GLY-121	A	4.5		ASN-294	D/A	37.5
	GLU-202	D	2.5		THR-293	A	9.5
	TRP-86	A	2		GLN-133	A	5
**JW7**	TYR-124	A	62.5		SER-386	A	7
	TYR-337	A	9.5		GLY-291	D	5.5
	SER-203	D	3.5		VAL-130	D	2.5
	HIS-447	D	2		ASP-93	D	2
**JW8**	PHE-295	A	67	**JW8**	TYR-132	D	9.5
	PHE-338	A	6		GLN-134	A	8
	TYR-337	A	2.5		LYS-168	D	7.5
	GLU-292	D	2.5		TYR-129	A	3
	SER-293	A	2.5				

**Table 3:** Pharmacokinetic parameters of the best-proposed inhibitors and reference compounds (DON—donepezil, QUE—quercetin, TAC—tacrine, and VER—verubecestat). D—H-bond donor; A—H-bond acceptor; H—high; L—low; M—moderate; Y—yes; N—no.

Ligand	H-Bond D/A	logP	Rat Acute Oral Toxicity	Human Carcinogenicity/ Hepa/Skin/Respiratory/ AMES/Eye-Toxicity	Lipiński/Pfizer/GSK/ Golden Triangle Rule	QED ^1^	Sa-Score ^2^	HIA ^3^/F_20%_ ^4^/ PPB ^5^/BBB ^6^/ PGPinh ^7^/PGBsub ^8^
**JW4**	2/6	4.026	L	L/L/H/L/M/L	Y/Y/N/Y	0.676	1.662	Y/Y/Y/N/N/Y
**JW7**	2/8	3.147	L	L/L/L/y/M/L	Y/Y/N/Y	0.549	1.667	Y/Y/Y/Y/N/N
**JW8**	2/6	3.957	M	L/L/H/L/M/L	Y/Y/Y/Y	0.676	1.618	Y/Y/Y/N/N/Y
DON	0/4	4.313	L	L/M/L/H/L/L	Y/N/N/Y	0.747	2.667	Y/Y/Y/Y/N/N
QUE	5/7	2.155	L	L/L/H/L/M/L	Y/Y/Y/Y	0.434	2.545	Y/N/Y/Y/N/N
TAC	2/2	2.739	L	H/M/H/H/H/L	Y/Y/Y/N	0.559	1.641	Y/N/Y/Y/N/Y
VER	3/8	1.603	H	M/H/M/H/L/L	Y/Y/N/Y	0.795	3.246	Y/Y/Y/Y/N/Y

**^1^** Drug-likeness (attractive > 0.67; unattractive 0.49–0.67; too complex < 0.34); **^2^** ease of synthesis (≥6 difficult; <6 easy); ^3^ **HIA**—human intestinal absorption; ^4^ F_20_**_%_**—20% bioavailability; **^5^ PPB**—plasma protein binding, not optimal; ^6^ **BBB**—blood–brain barrier penetration; ^7^ **PGPinh—PGP** inhibitor; ^8^ **PGBsub—PGB** substrate.

## Data Availability

Not applicable.

## Supplementary Materials

The following supporting information can be downloaded at: https://www.mdpi.com/article/10.3390/ijms241914901/s1.
