# Supplementary material for: New Benzamides as Multi-Targeted Compounds: A Study on Synthesis, AChE and BACE1 Inhibitory Activity and Molecular Docking
Source: Int J Mol Sci. 2023 Oct 4;24(19):14901. doi: 10.3390/ijms241914901 (PMC10573752; doi:10.3390/ijms241914901)

1H NMR spectrum of compound ***N,N'*-(1,3-phenylene)bis(3,4,5-trimethoxybenzamide) JW1**

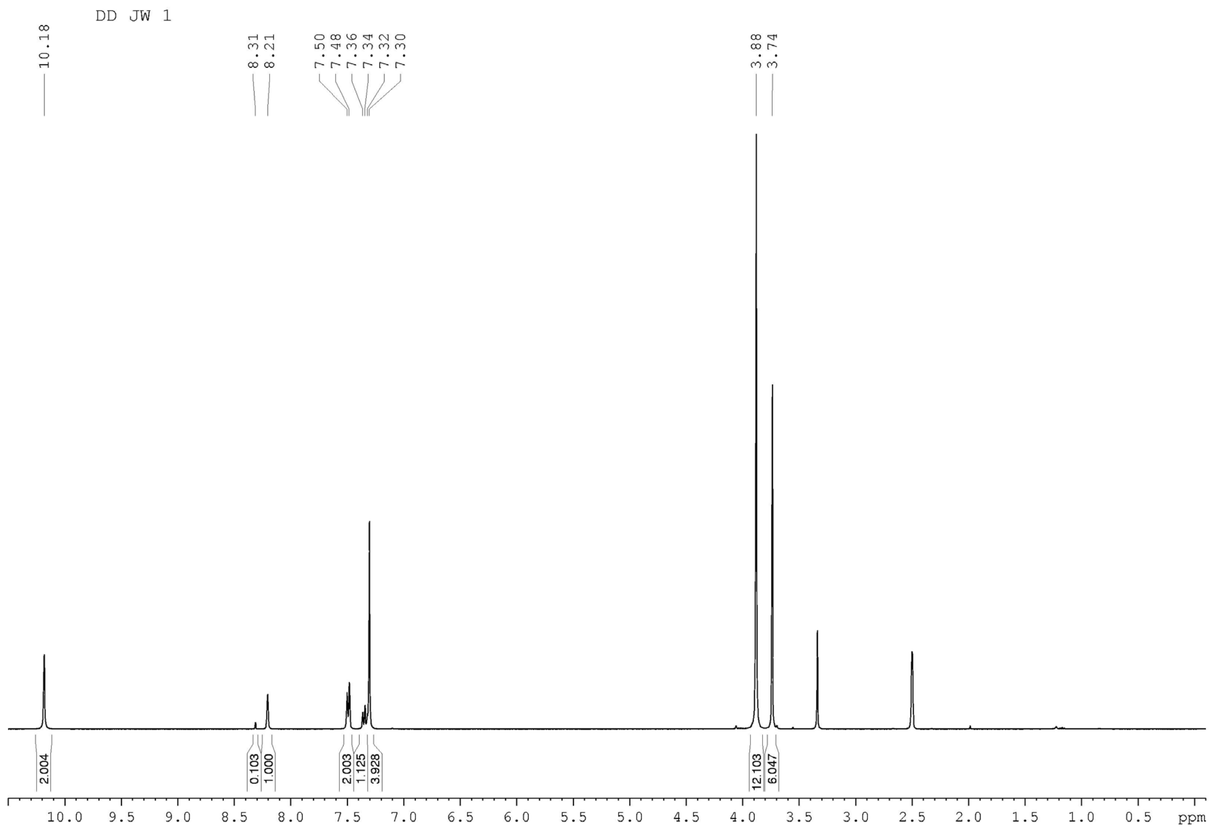

13C NMR spectrum of compound ***N,N'*-(1,3-phenylene)bis(3,4,5-trimethoxybenzamide) JW1**

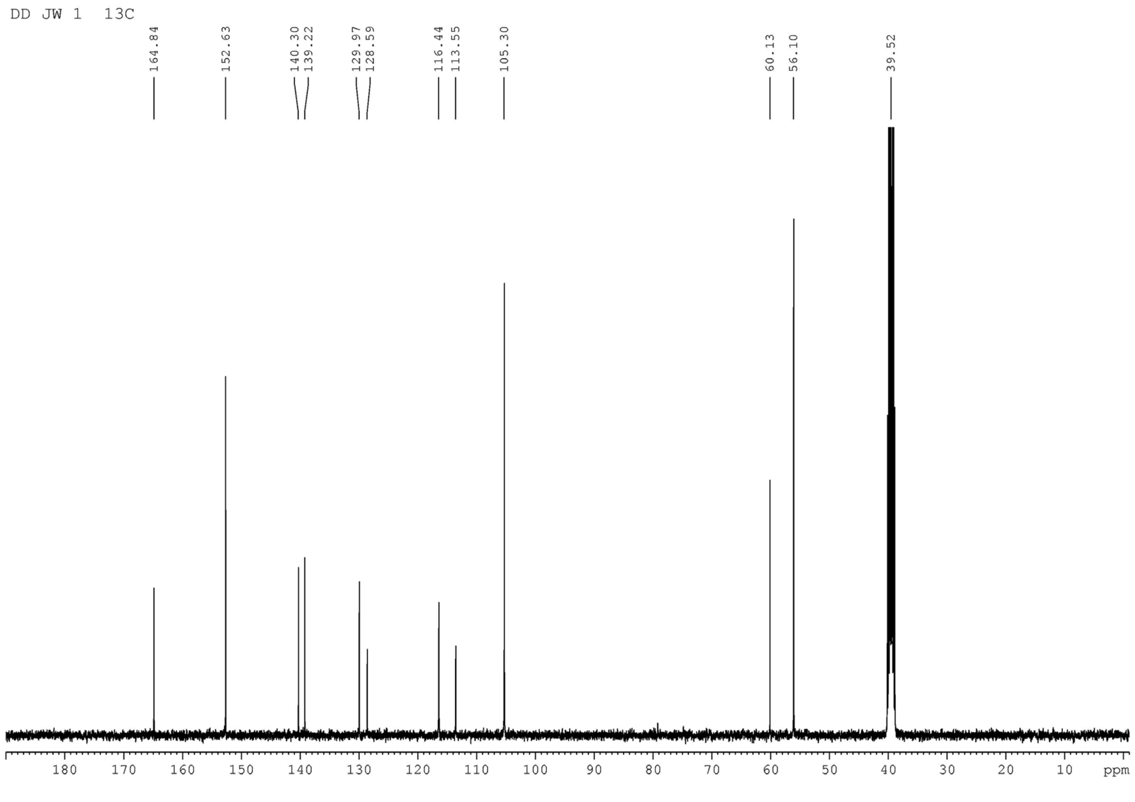

1H NMR spectrum of compound ***N,N'*-(1,3-phenylene)bis(3,5-dimethoxybenzamide) JW2**

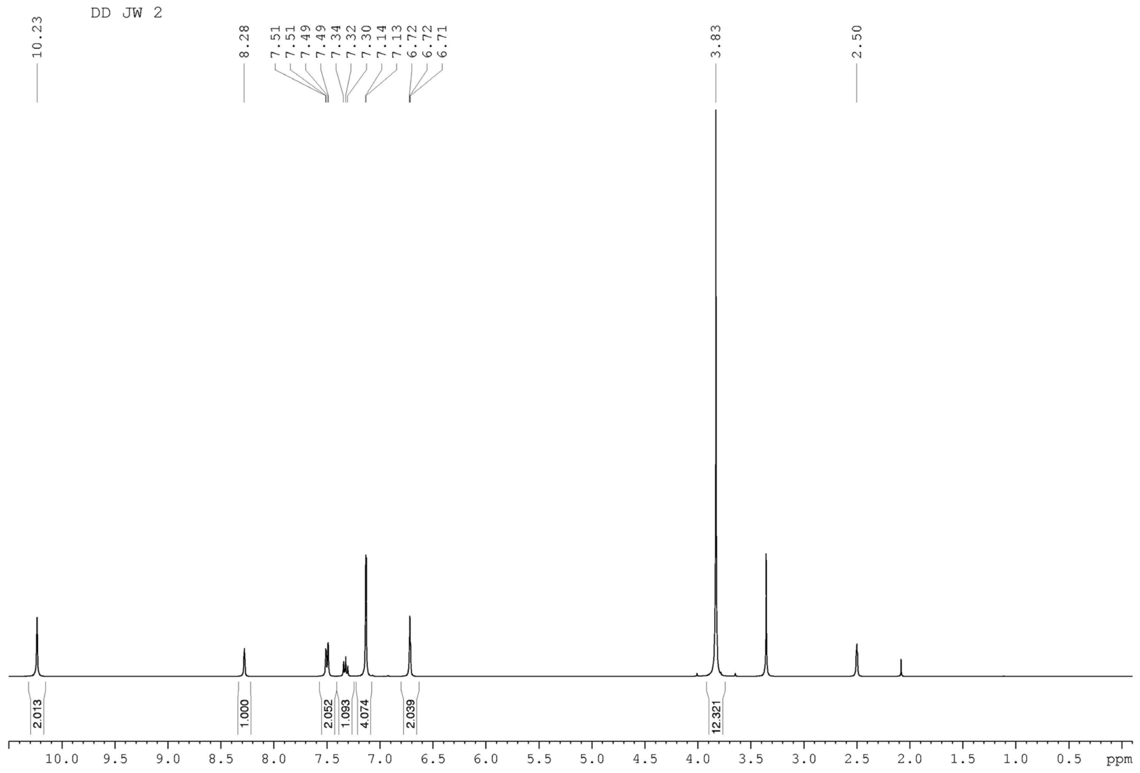

13C NMR spectrum of compound ***N,N'*-(1,3-phenylene)bis(3,5-dimethoxybenzamide) JW2**

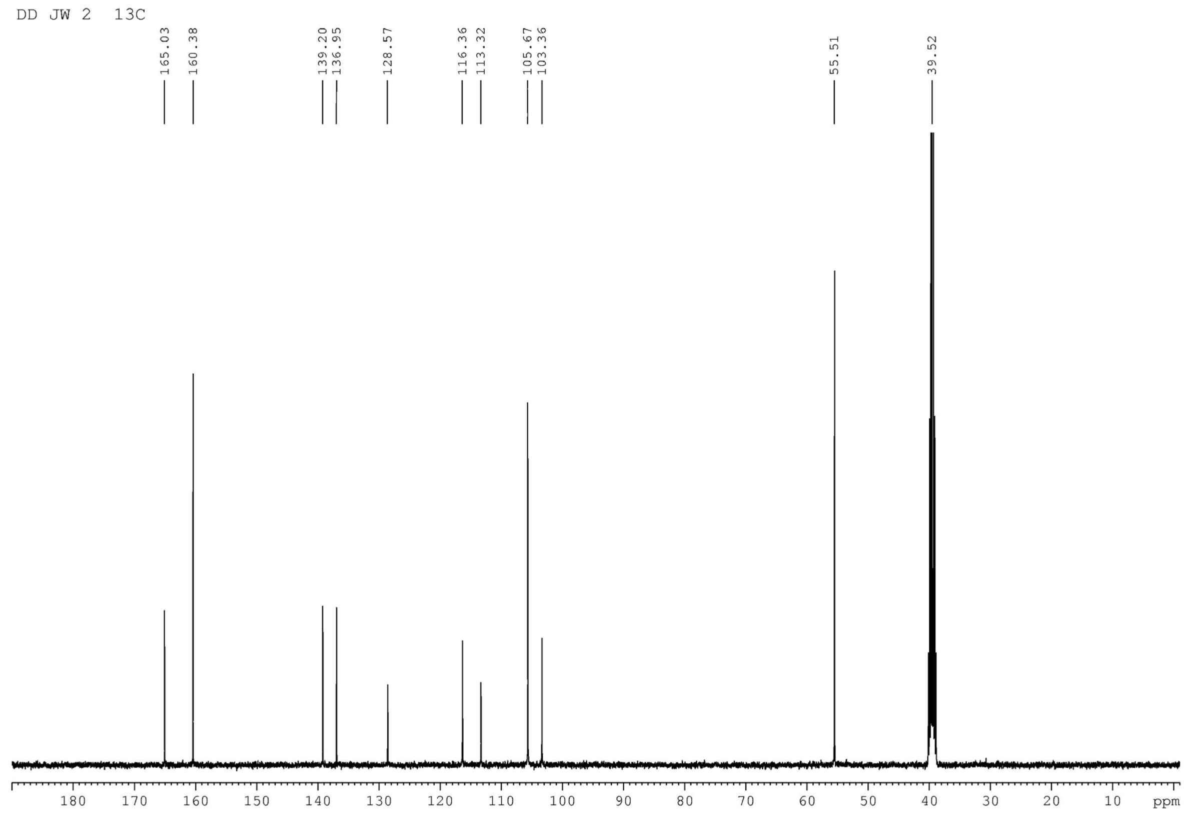

1H NMR spectrum of compound **3,4-dimethoxy-*N*-[3-[(3,4-dimethoxybenzyl)amino]phenyl]benzamide JW3**

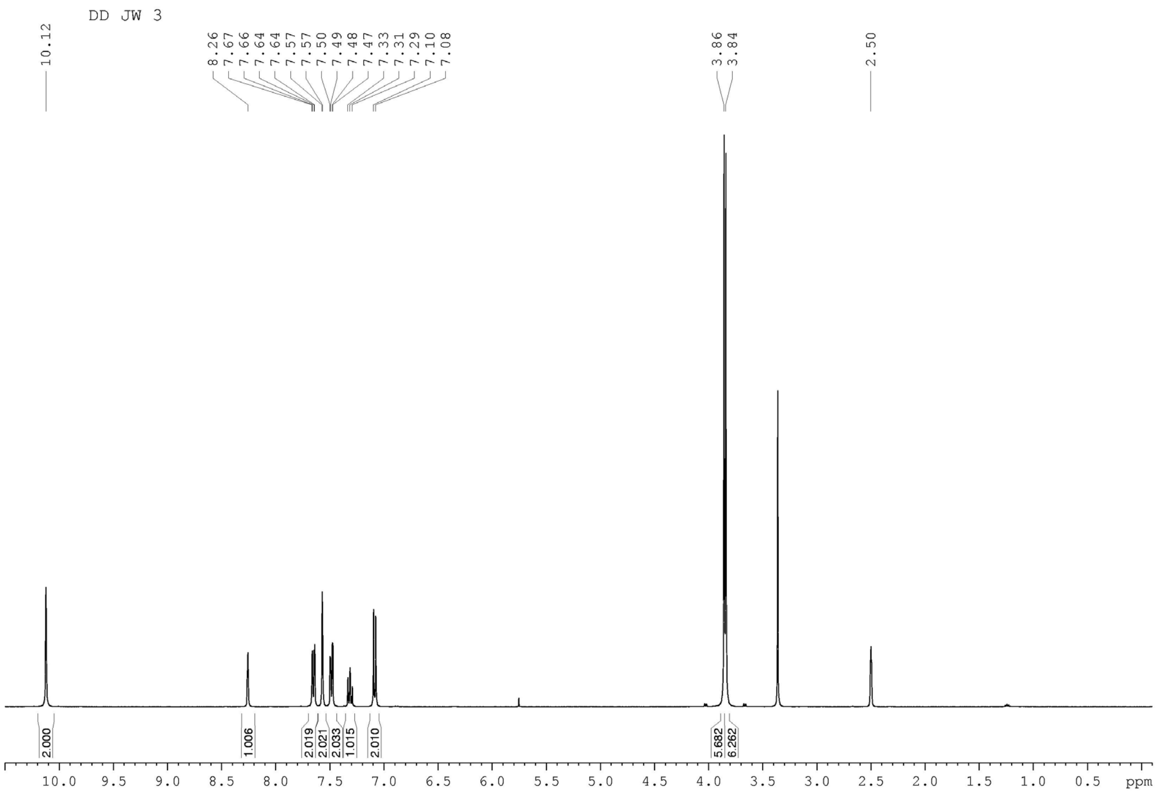

13C NMR spectrum of compound **3,4-dimethoxy-*N*-[3-[(3,4-dimethoxybenzyl)amino]phenyl]benzamide JW3**

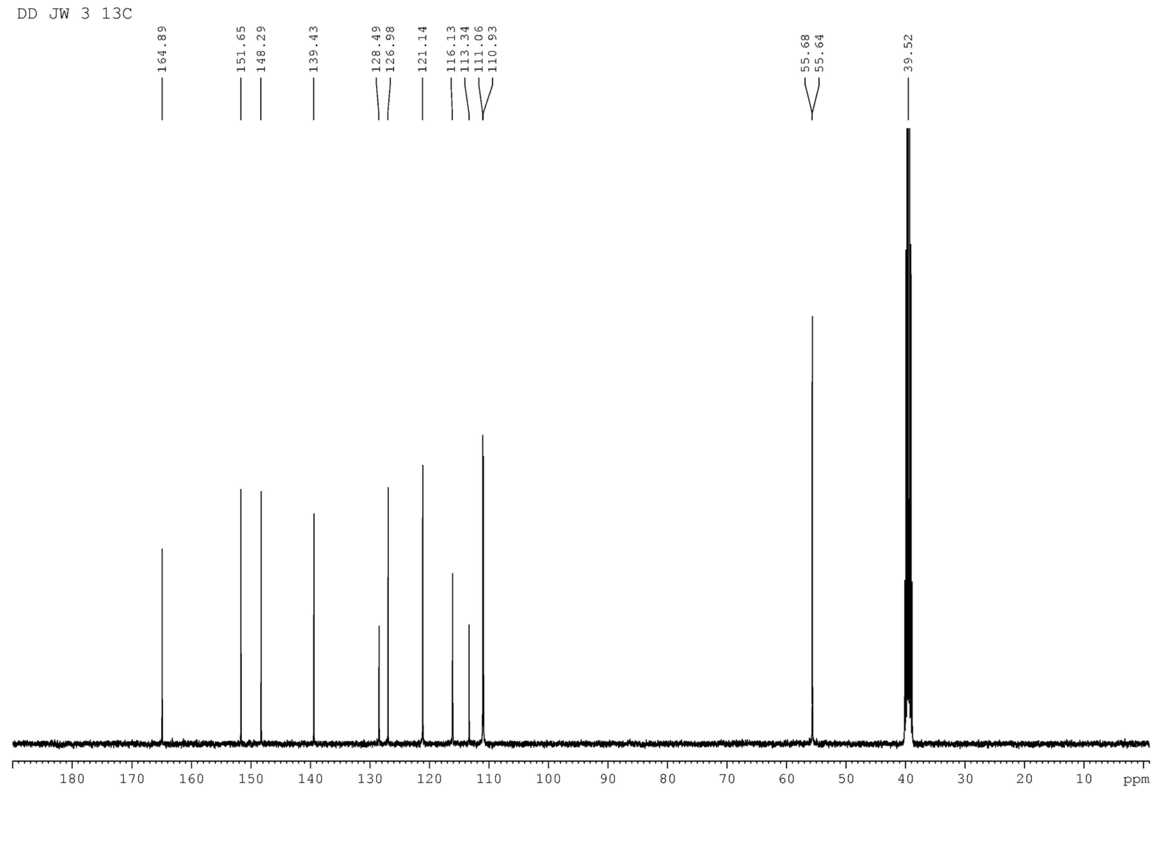

1H NMR spectrum of compound **3-methoxy-*N*-[3-[(3,4-dimethoxybenzyl)amino]phenyl]benzamide JW4**

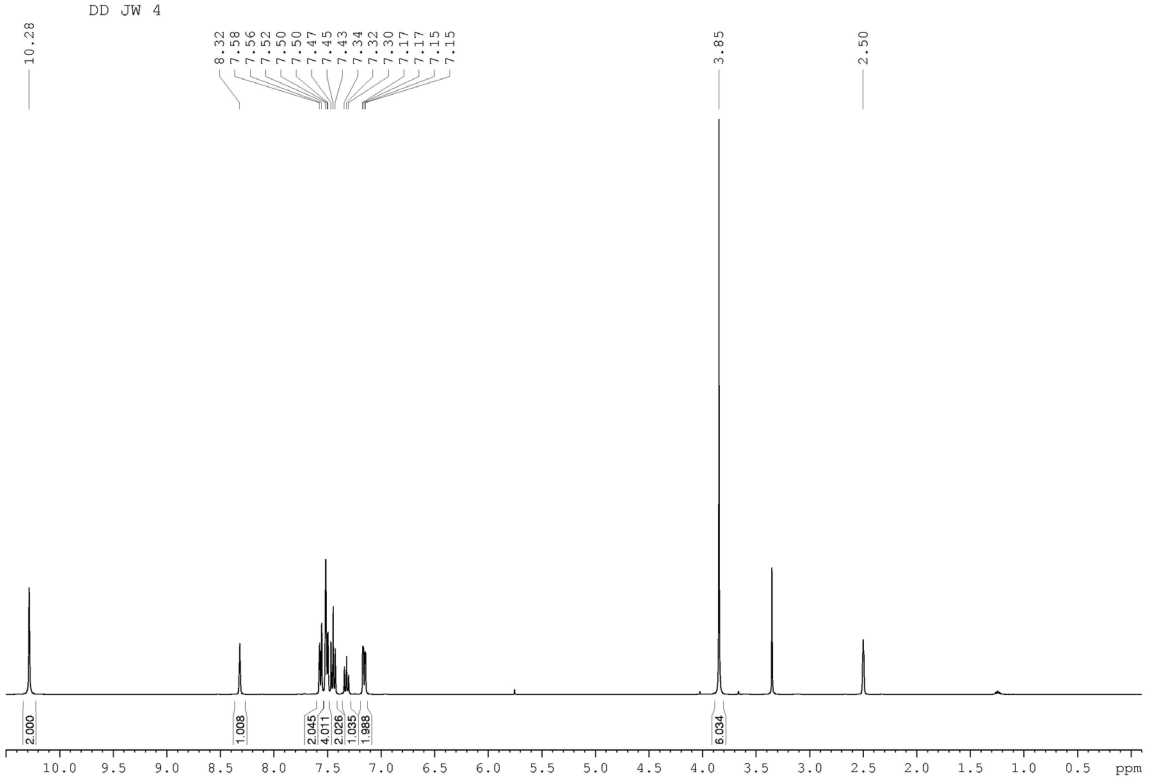

13C NMR spectrum of compound **3-methoxy-*N*-[3-[(3,4-dimethoxybenzyl)amino]phenyl]benzamide JW4**

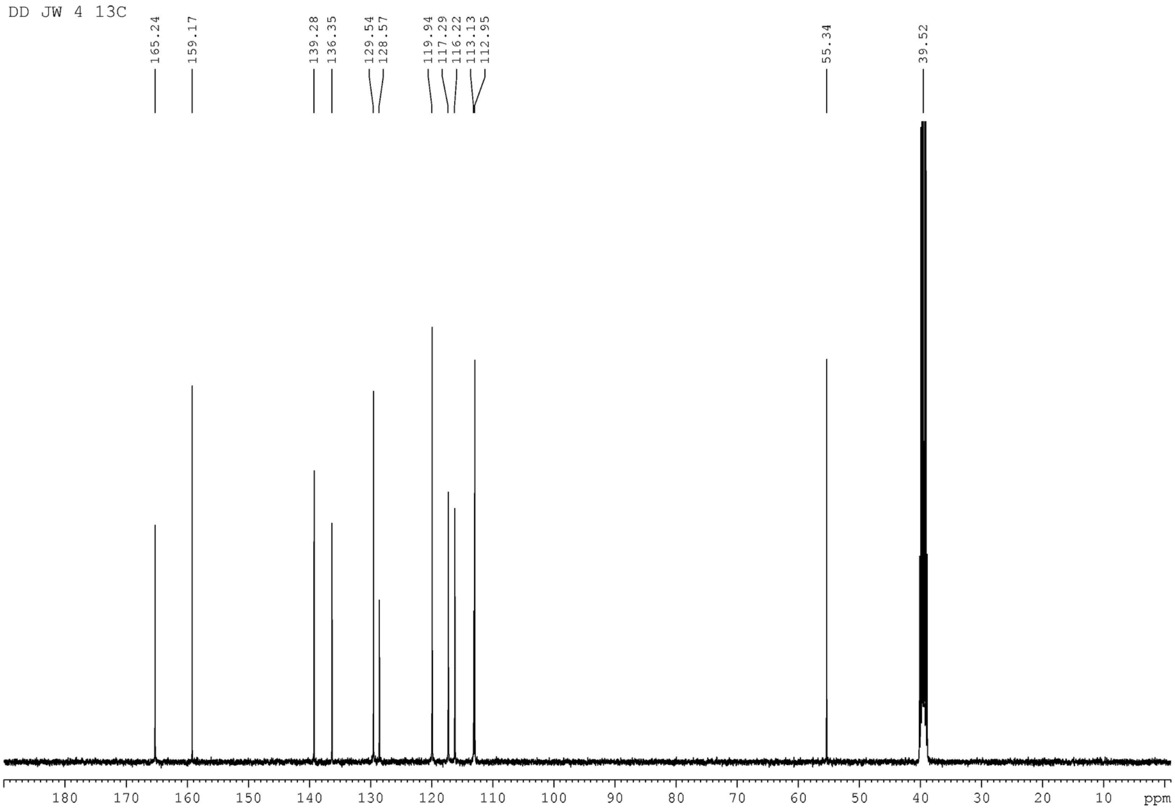

1H NMR spectrum of compound ***N*,*N'*-(1,4-phenylene)bis(3,4,5-trimethoxybenzamide) JW5**

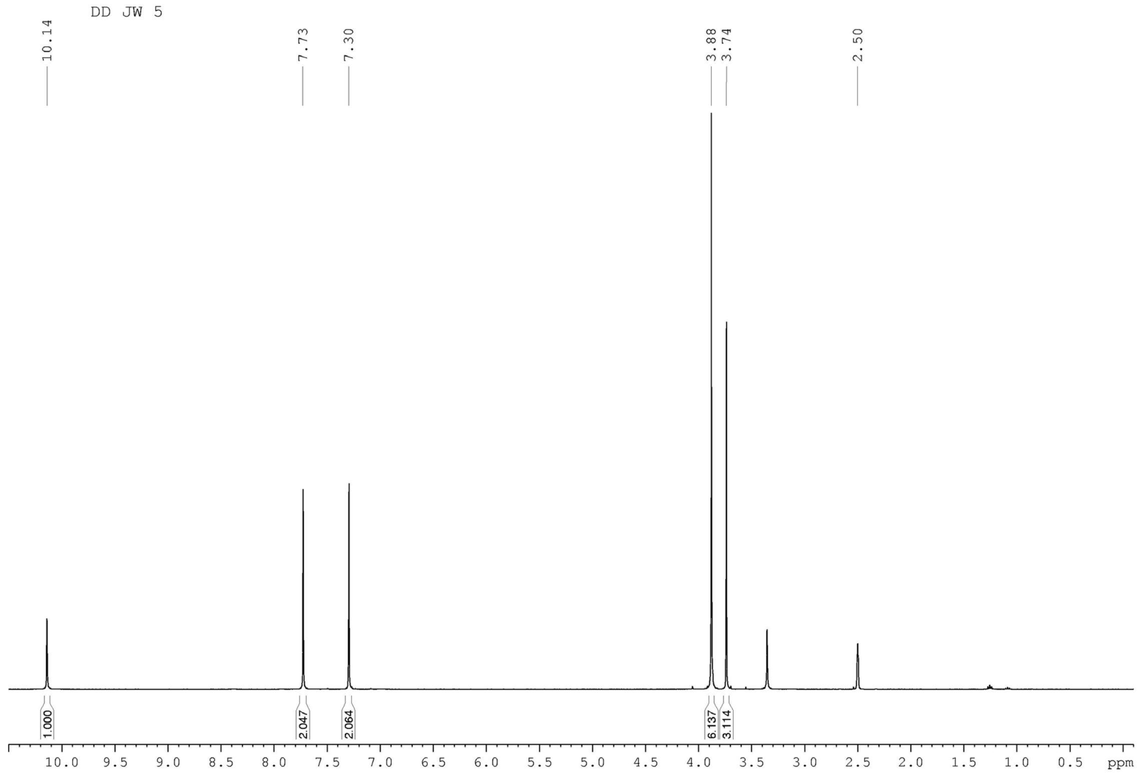

13C NMR spectrum of compound ***N*,*N'*-(1,4-phenylene)bis(3,4,5-trimethoxybenzamide) JW5**

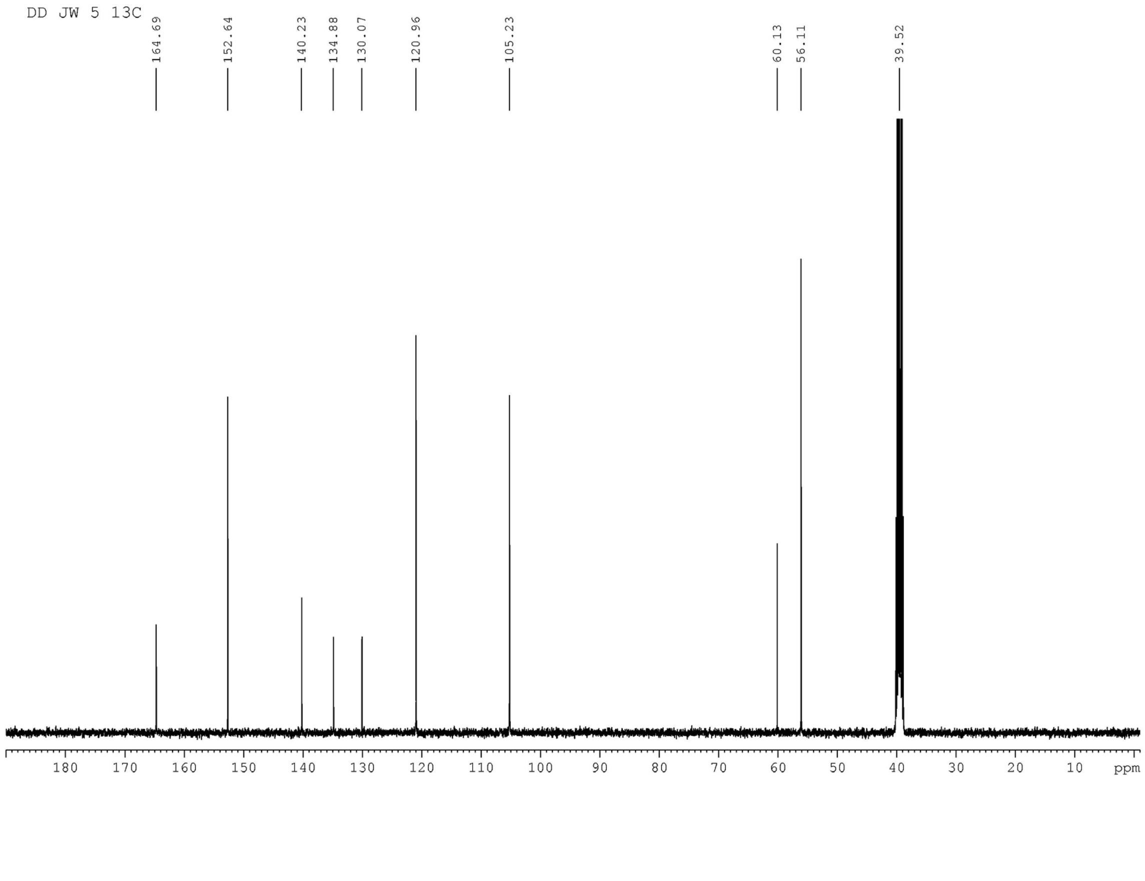

1H NMR spectrum of compound ***N,N'*-(1,4-phenylene)bis(3,5-dimethoxybenzamide) JW6**

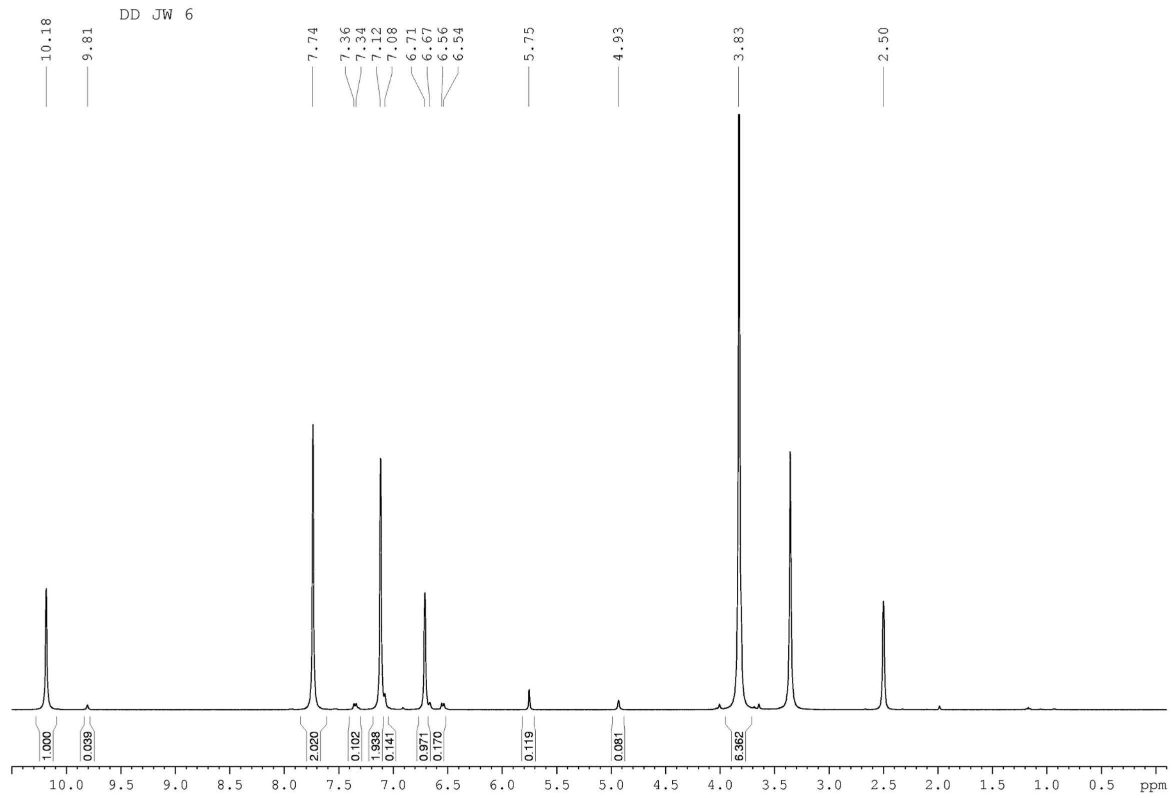

13C NMR spectrum of compound ***N,N'*-(1,4-phenylene)bis(3,5-dimethoxybenzamide) JW6**

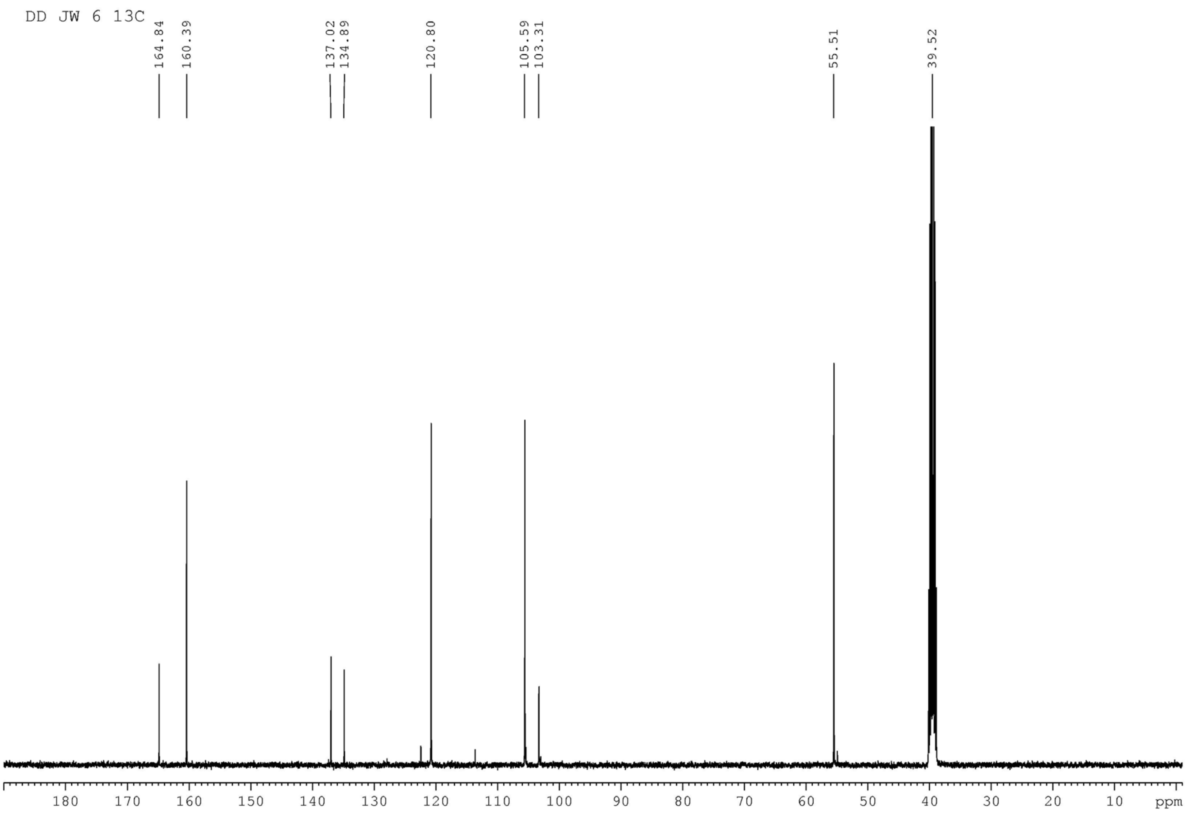

1H NMR spectrum of compound ***N,N'*-(1,4-phenylene)bis(3,4-dimethoxybenzamide) JW7**

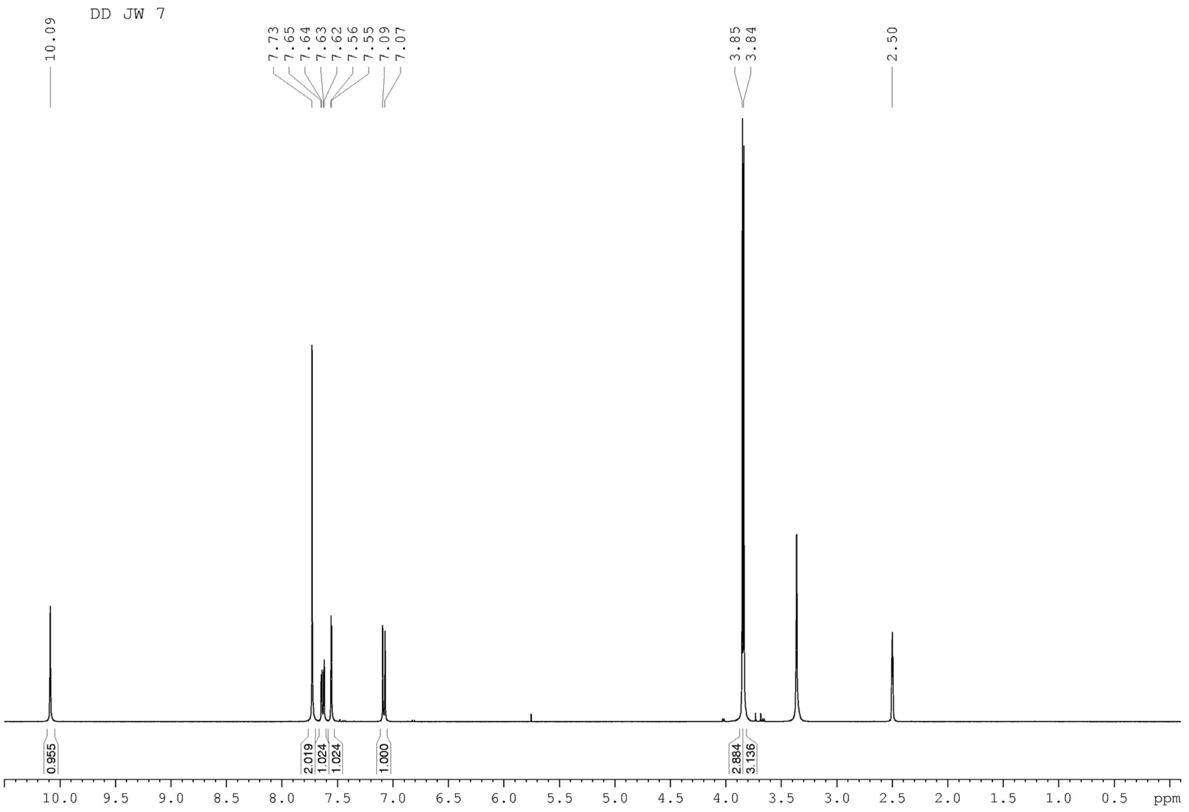

13C NMR spectrum of compound ***N,N'*-(1,4-phenylene)bis(3,4-dimethoxybenzamide) JW7**

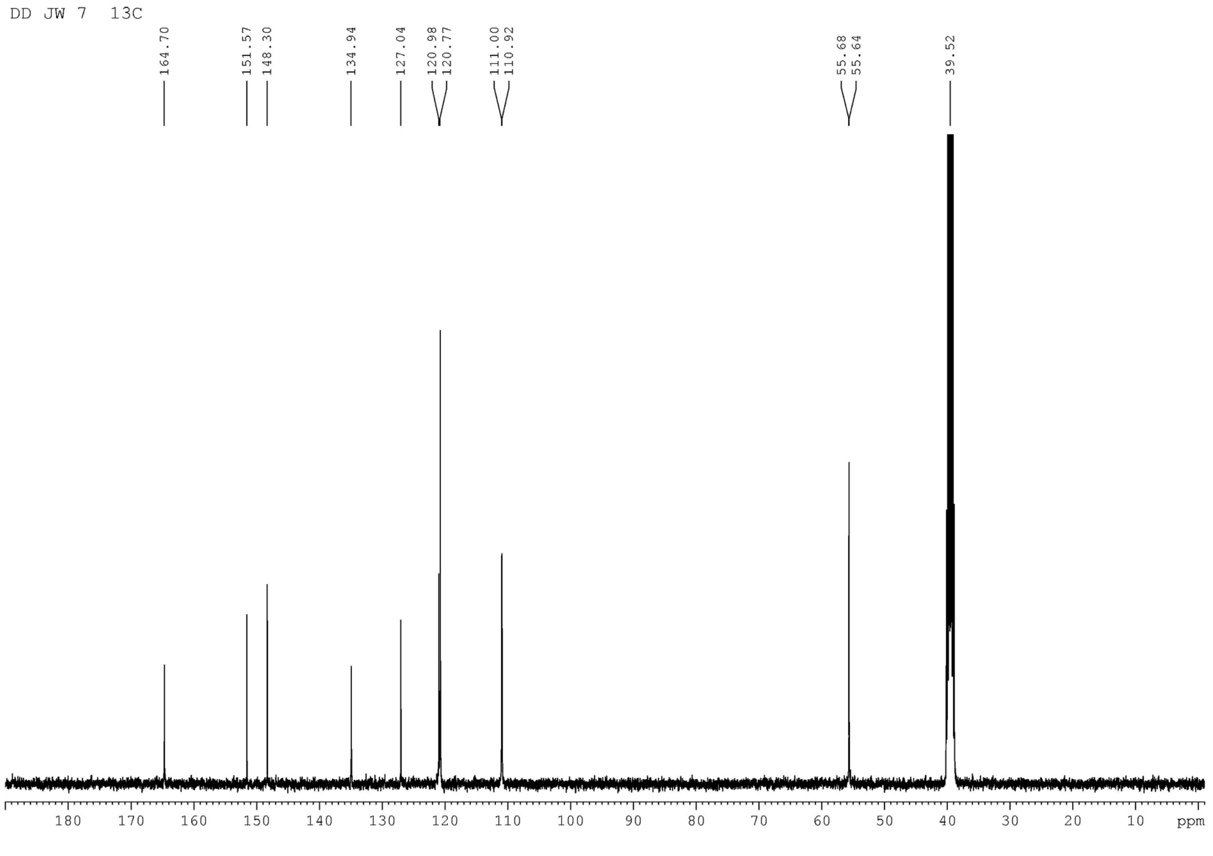

1H NMR spectrum of compound ***N,N'*-(1,4-phenylene)bis(3-methoxybenzamide) JW8**

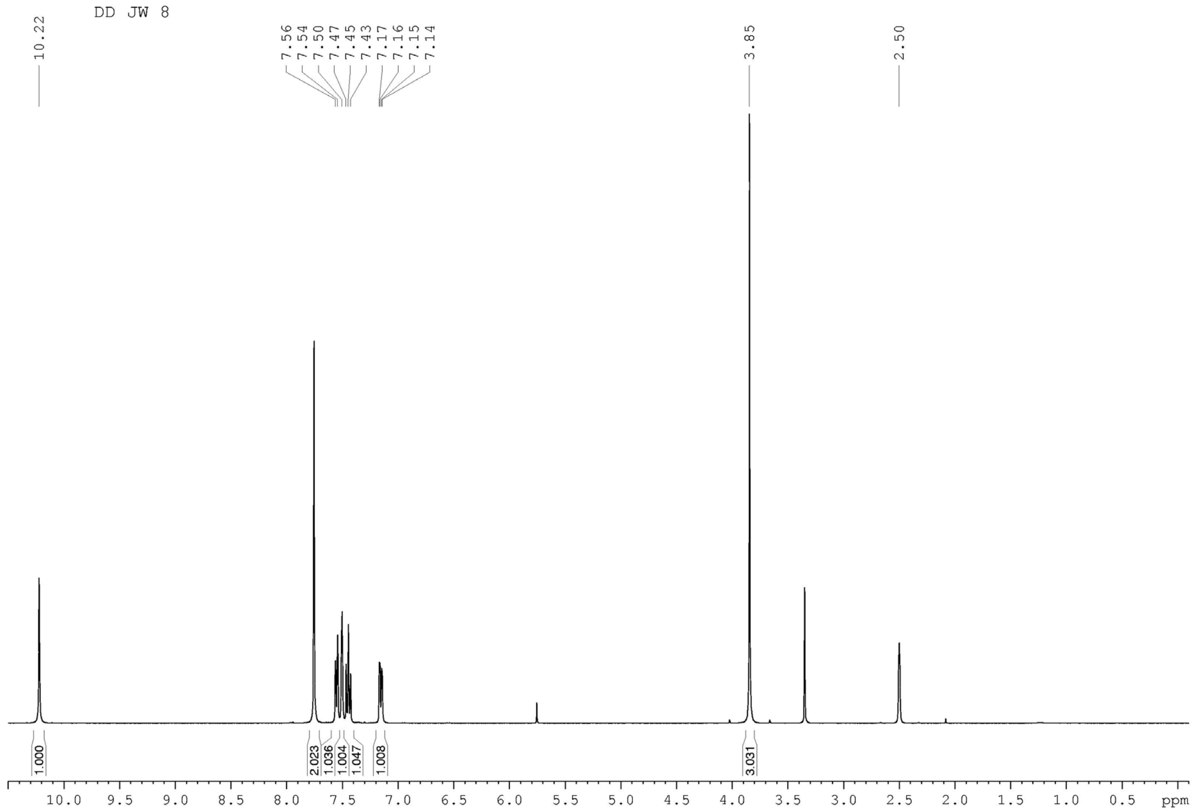

13C NMR spectrum of compound ***N,N'*-(1,4-phenylene)bis(3-methoxybenzamide) JW8**

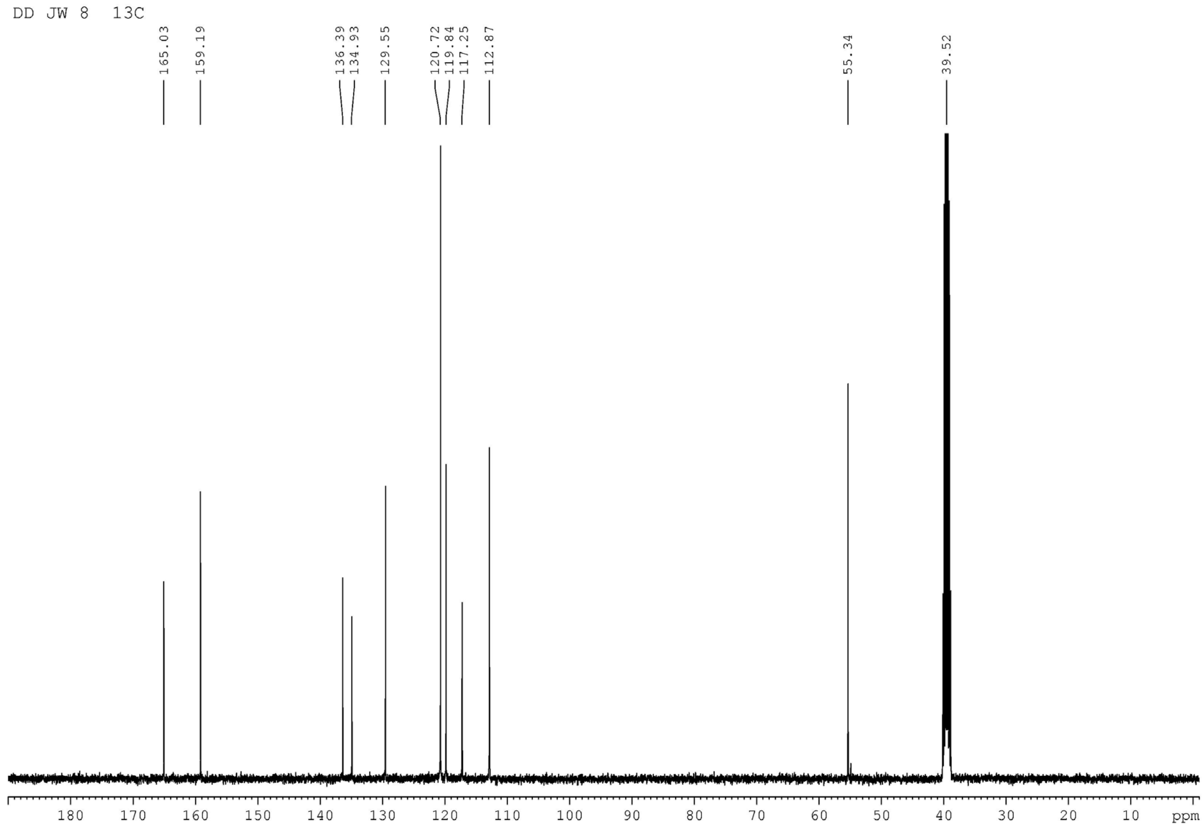

1H NMR spectrum of compound **3,4,5-Trimethoxy-*N*-(3-nitrophenyl)benzamide MB1**

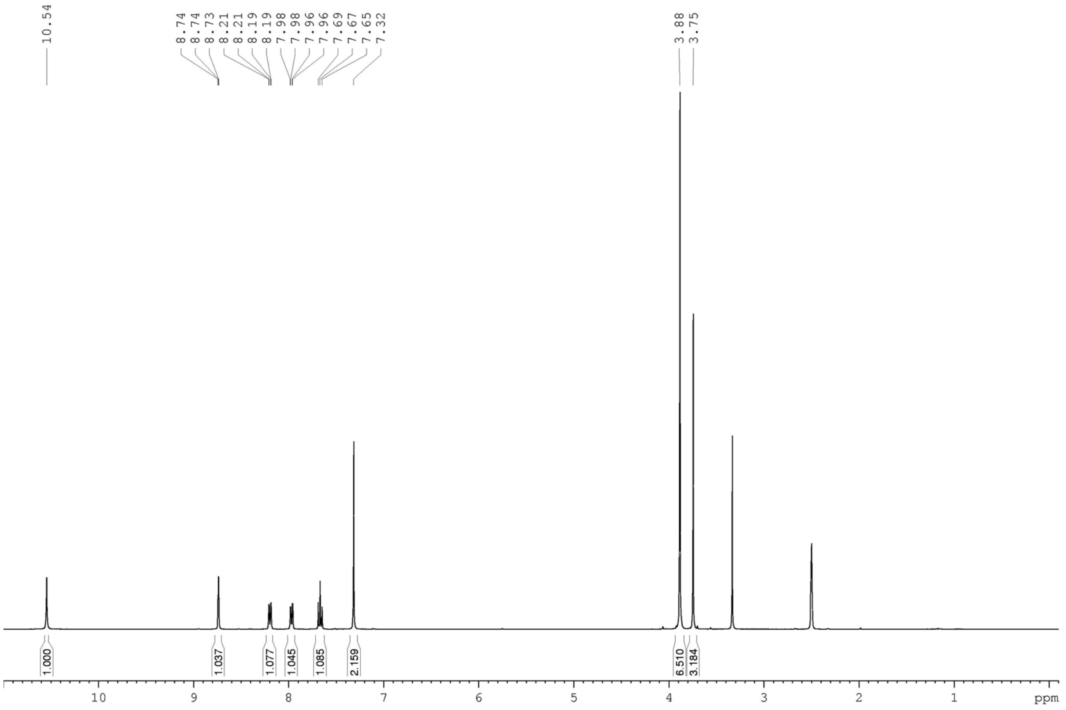

13C NMR spectrum of compound **3,4,5-Trimethoxy-*N*-(3-nitrophenyl)benzamide MB1**

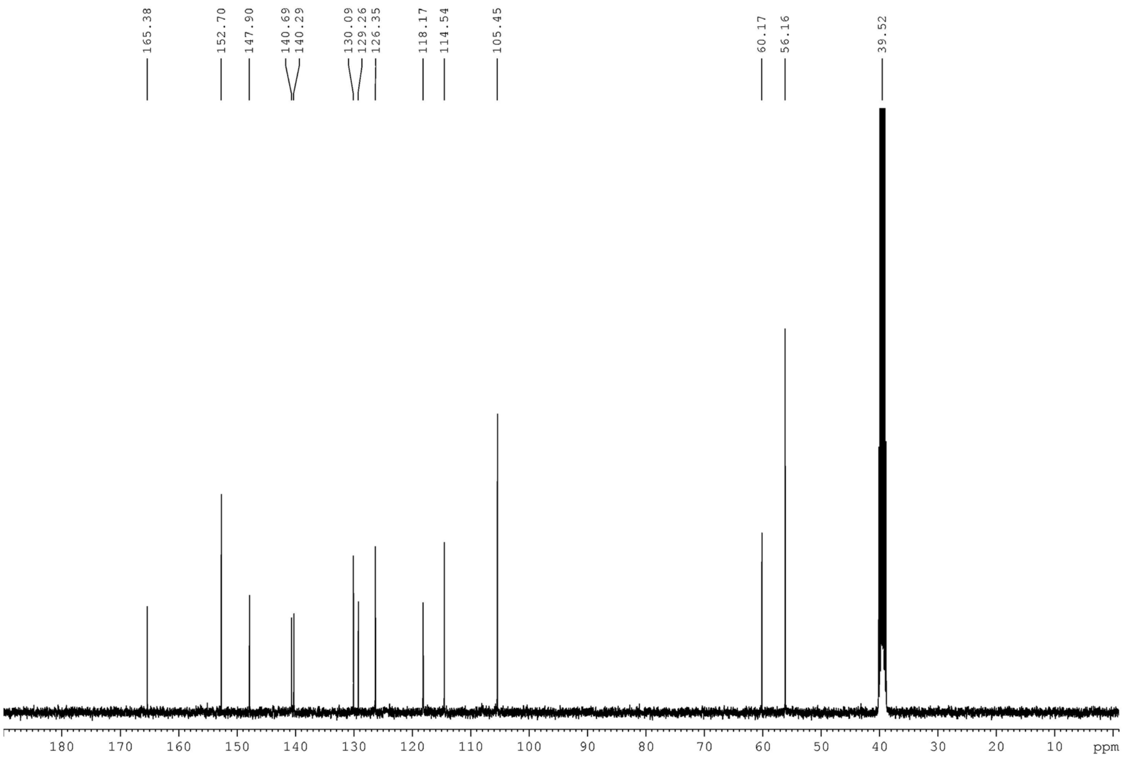

1H NMR spectrum of compound **3,4,5-Trimethoxy-*N*-(3,4,5-trimethoxyphenyl)benzamide MB3**

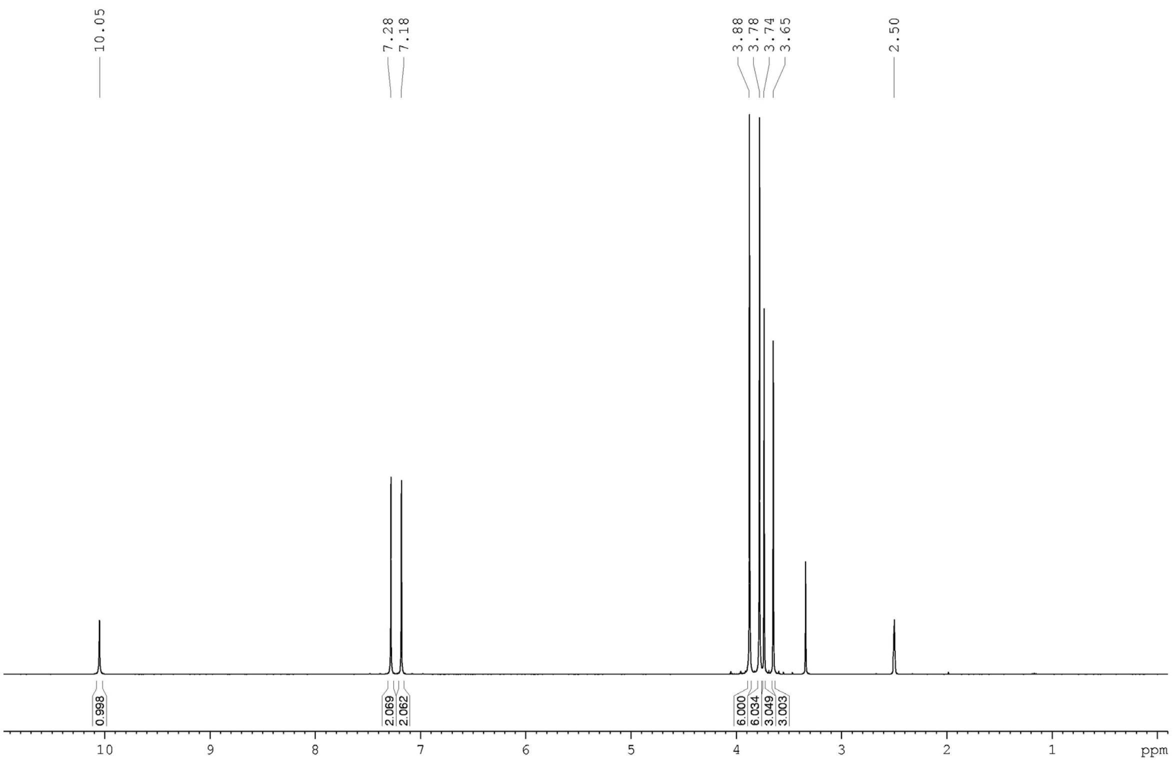

13C NMR spectrum of compound **3,4,5-Trimethoxy-*N*-(3,4,5-trimethoxyphenyl)benzamide MB3**

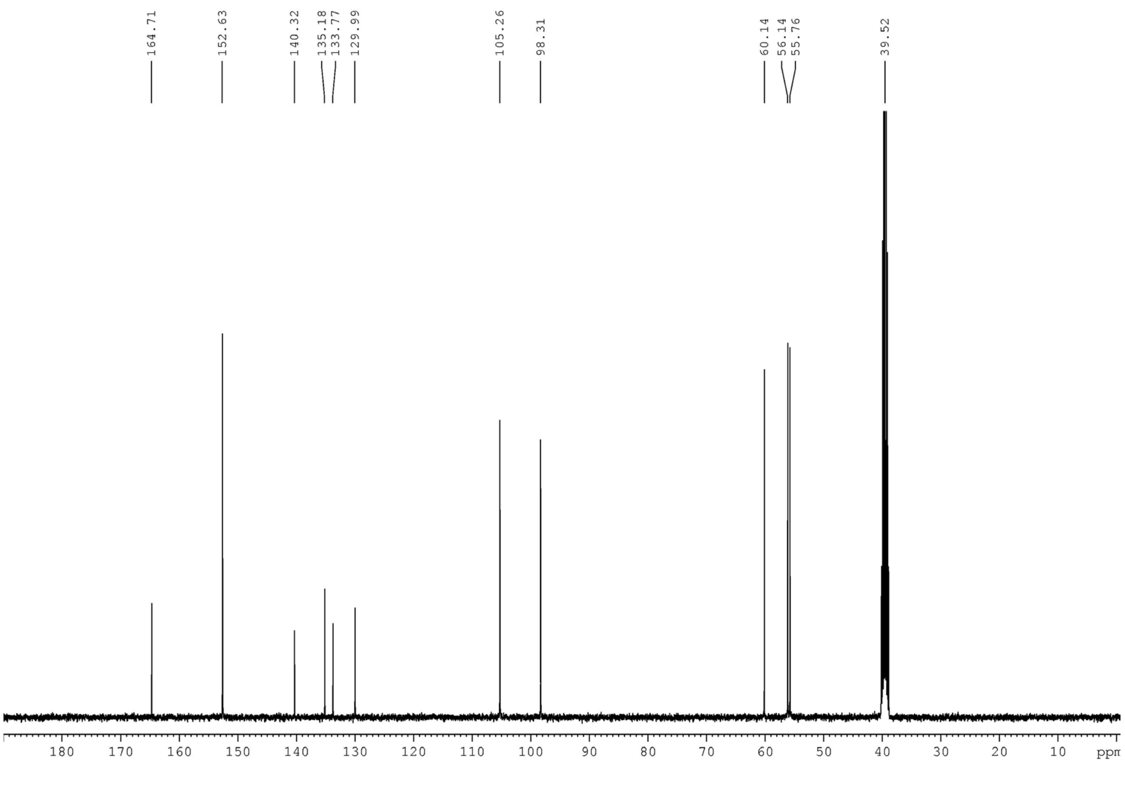

1H NMR spectrum of compound **3,4,5-Trimethoxy-*N*-(3,4,5-trimethoxybenzyl)benzamide MB4**

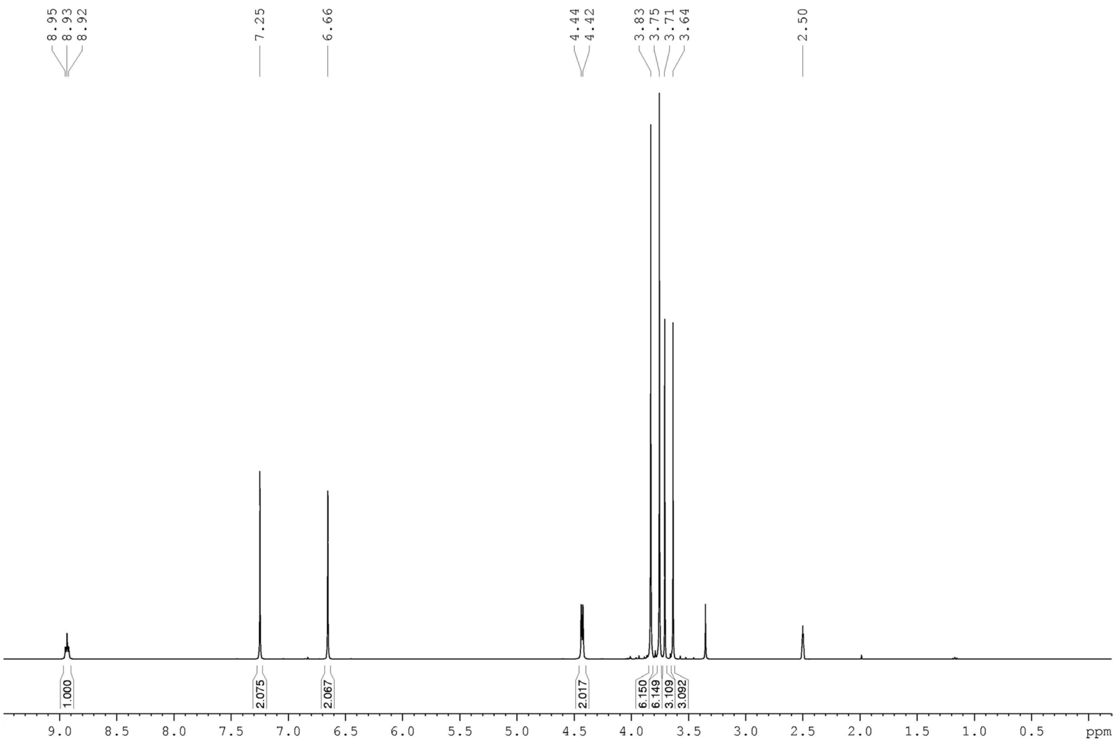

13C NMR spectrum of compound **3,4,5-Trimethoxy-*N*-(3,4,5-trimethoxybenzyl)benzamide MB4**

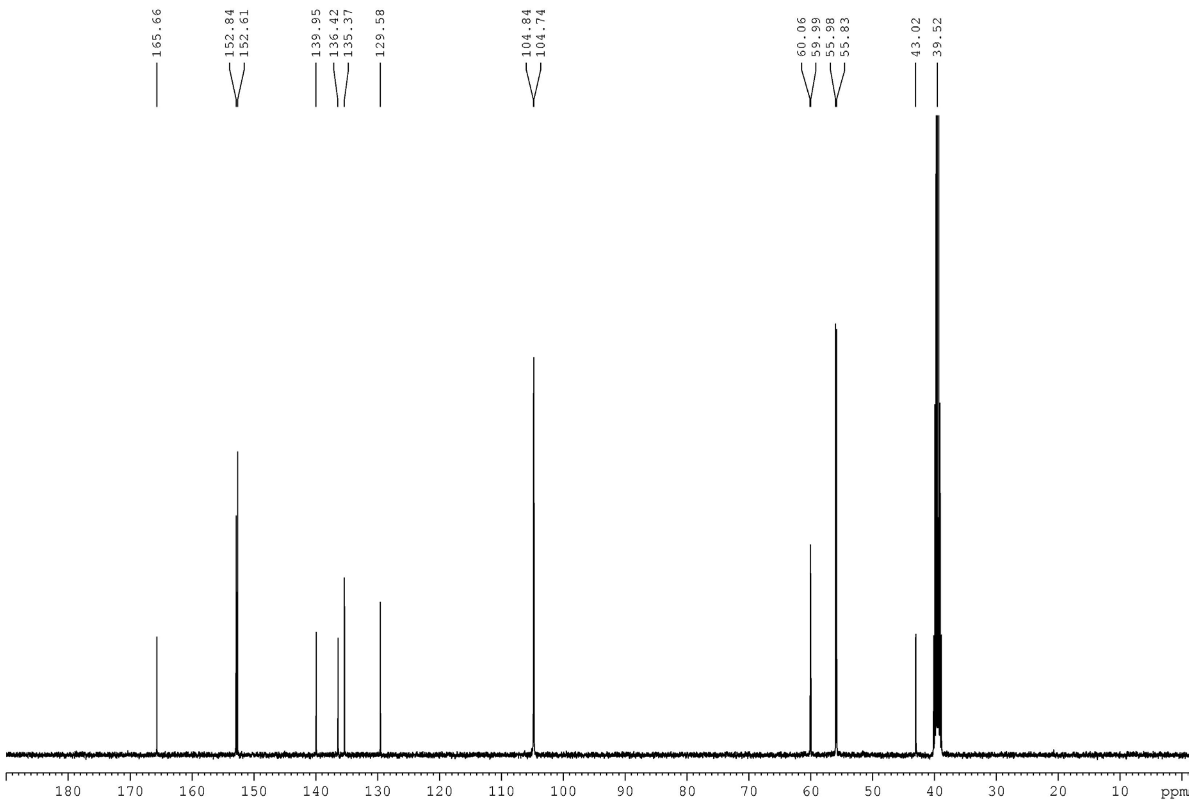

Supplement: Supplementary file 1 [file ijms-24-14901-s001.zip › Supporting_NMR_spectra.pdf]
