# Supplementary material for: New Benzamides as Multi-Targeted Compounds: A Study on Synthesis, AChE and BACE1 Inhibitory Activity and Molecular Docking
Source: Int J Mol Sci. 2023 Oct 4;24(19):14901. doi: 10.3390/ijms241914901 (PMC10573752; doi:10.3390/ijms241914901)
Supplement: Supplementary file 1 [file ijms-24-14901-s001.zip › Table S1 and S2.pdf]

Table S1. Statistical parameters of 20-ns molecular dynamics simulation of AChE and its complexes. Average - arithmetic mean, SD - standard deviation, SD/average - coefficient of variation. Averaged over ligands – arithmetic mean from columns Donepezil, Tacrine, JW4, JW7, and JW8.

AChE								Averaged over ligands
Complex		APO	Donepezil	Tacrine	JW4	JW7	JW8	
**RMSD**	Average	1.6	1.5	1.4	1.5	1.5	1.4	**1.45**
	SD	0.20	0.20	0.13	0.20	0.19	0.14	**0.17**
	SD/average	0.126	0.134	0.098	0.131	0.129	0.096	**0.12**
**SASA**	Average	22355.6	22424.8	22131.7	22232.6	22317.6	22087.0	**22238.75**
	SD	459.89	368.96	217.27	354.18	301.64	308.83	**310.18**
	SD/average	0.021	0.016	0.010	0.016	0.014	0.014	**0.014**
**Rg**	Average	23.1	23.0	23.0	23.0	23.1	23.0	**23.01**
	SD	0.10	0.09	0.07	0.09	0.10	0.07	**0.08**
	SD/average	0.004	0.004	0.003	0.004	0.004	0.003	**0.004**
**RMSF**	Average	2.74	0.76	0.74	0.76	0.74	0.77	**0.76**
	SD	0.72	0.44	0.37	0.42	0.37	0.45	**0.41**
	SD/average	0.26	0.58	0.50	0.56	0.50	0.59	**0.55**

Table S2. Statistical parameters of 20-ns molecular dynamics simulation of BACE1 and its complexes. Average - arithmetic mean, SD - standard deviation, SD/average - coefficient of variation. Averaged over ligands – arithmetic mean from columns Verubecestat, Quercentine, JW4, JW7, and JW8.

BACE1								Averaged over ligands
Complex		APO	Verubecestat	Quercentine	JW4	JW7	JW8	
**RMSD**	Average	1.94	1.62	1.76	1.28	1.74	1.55	**1.59**
	SD	0.26	0.22	0.25	0.18	0.27	0.22	**0.23**
	SD/average	0.13	0.13	0.14	0.14	0.15	0.14	**0.14**
**SASA**	Average	18845.6	18352.4	18540.4	18484.2	18882.5	18195.9	**18491.08**
	SD	284.5	257.3	380.8	331.3	340.0	277.9	**317.48**
	SD/average	0.015	0.014	0.021	0.018	0.018	0.015	**0.02**
**Rg**	Average	21.6	21.3	21.4	21.2	21.4	21.2	**21.32**
	SD	0.13	0.11	0.15	0.14	0.19	0.13	**0.15**
	SD/average	0.006	0.005	0.007	0.006	0.009	0.006	**0.007**
**RMSF**	Average	0.79	0.77	0.85	1.77	0.91	0.80	**1.02**
	SD	0.36	0.38	0.41	0.34	0.48	0.45	**0.41**
	SD/average	0.45	0.49	0.49	0.19	0.52	0.55	**0.45**
